# Evidence on Technology-Based Psychological Interventions in Diagnosed Depression: Systematic Review

**DOI:** 10.2196/21700

**Published:** 2021-02-10

**Authors:** Moritz Köhnen, Mareike Dreier, Tharanya Seeralan, Levente Kriston, Martin Härter, Harald Baumeister, Sarah Liebherz

**Affiliations:** 1 Department of Medical Psychology University Medical Center Hamburg-Eppendorf Hamburg Germany; 2 Department for Clinical Psychology and Psychotherapy Ulm University Ulm Germany

**Keywords:** internet, telephone, psychotherapy, depression, depressive disorder, systematic review, mobile phone

## Abstract

**Background:**

Evidence on technology-based psychological interventions (TBIs) for the treatment of depression is rapidly growing and covers a broad scope of research. Despite extensive research in this field, guideline recommendations are still limited to the general effectiveness of TBIs.

**Objective:**

This study aims to structure evidence on TBIs by considering different application areas (eg, TBIs for acute treatment and their implementation in health care, such as stand-alone interventions) and treatment characteristics (eg, therapeutic rationale of TBIs) to provide a comprehensive evidence base and to identify research gaps in TBIs for diagnosed depression. Moreover, the reporting of negative events in the included studies is investigated in this review to enable subsequent safety assessment of the TBIs.

**Methods:**

Randomized controlled trials on adults diagnosed with unipolar depression receiving any kind of psychotherapeutic treatment, which was at least partly delivered by a technical medium, were eligible for inclusion in our preregistered systematic review. We searched for trials in CENTRAL (Cochrane Central Register of Controlled Trials; until August 2020), MEDLINE, PsycINFO, PSYNDEX, CINAHL; until the end of January 2018), clinical trial registers, and sources of gray literature (until the end of January 2019). Study selection and data extraction were conducted by 2 review authors independently.

**Results:**

Database searches resulted in 15,546 records, of which 241 publications were included, representing 83 completed studies and 60 studies awaiting classification (ie, preregistered studies, study protocols). Almost all completed studies (78/83, 94%) addressed the acute treatment phase, being largely either implemented as stand-alone interventions (66/83, 80%) or blended treatment approaches (12/83, 14%). Studies on TBIs for aftercare (4/83, 5%) and for bridging waiting periods (1/83, 1%) were scarce. Most TBI study arms (n=107) were guided (59/107, 55.1%), delivered via the internet (80/107, 74.8%), and based on cognitive behavioral treatment approaches (88/107, 79.4%). Almost all studies (77/83, 93%) reported information on negative events, considering dropouts from treatment as a negative event. However, reports on negative events were heterogeneous and largely unsystematic.

**Conclusions:**

Research has given little attention to studies evaluating TBIs for aftercare and for bridging waiting periods in people with depression, even though TBIs are seen as highly promising in these application areas; thus, high quality studies are urgently needed. In addition, the variety of therapeutic rationales on TBIs has barely been represented by identified studies hindering the consideration of patient preferences when planning treatment. Finally, future studies should use specific guidelines to systematically assess and report negative events.

**Trial Registration:**

International Prospective Register of Systematic Reviews (PROSPERO) CRD42016050413; https://www.crd.york.ac.uk/prospero/display_record.php?ID=CRD42016050413.

**International Registered Report Identifier (IRRID):**

RR2-10.1136/bmjopen-2018-028042

## Introduction

Depression is a common [1] and debilitating mental disorder for both affected individuals and society. It is often accompanied by psychosocial difficulties [2], increased mortality [3], concurrent psychological [4] and/or somatic disorders [5], and high societal costs [6]. There are many effective treatment options for people with unipolar depression, especially psychotherapeutic (eg, cognitive behavioral therapy [CBT], interpersonal therapy) and pharmacological treatments [1,7]. Despite the high prevalence, burden, and the presence of many effective treatment options, depression is still underrecognized [8] and undertreated [9]. For example, in Germany—with a comparatively well-developed mental health care system—only 54% of people with a lifetime diagnosis of major depression and 62% with dysthymia report lifetime service use, indicating barriers and gaps in the health care system [10]. Technology-based psychological interventions (TBIs) are one option to address barriers (eg, long waiting periods before starting a treatment) and gaps (eg, providing psychotherapeutic treatment in rural areas) in the context of mental health care [11]. We defined TBIs as psychotherapeutic or psychological interventions being (at least partly) delivered by technical mediums and tailored to the treatment of depression (eg, guided or unguided web-based self-help programs, telephone therapy, or the combination of onsite therapy and web-based self-help; see study protocol by Köhnen et al [12] for details).

TBIs cover a heterogeneous group of treatments, differing in various aspects, as described by Ebert et al [13]: technical aspects (ie, the application of different technologies such as email or telephone), the amount of human support (eg, TBIs with or without human support, using either synchronous or asynchronous communication), theoretical background (ie, TBIs can be based on different therapeutic rationales), and application areas (eg, TBIs can be provided in different clinical phases of depression management).

In the last decade, research on TBIs has grown rapidly [14], resulting in many randomized controlled trials (RCTs) on people with depression [15-17] as well as systematic reviews [18-20]. Despite extensive research efforts in the field of TBIs for depression treatment, there are still neglected issues, which we aim to address in our systematic review.

First, there is no systematic review that structures available evidence on TBIs regarding different clinical phases of depression management (considering waiting periods, acute treatment, and aftercare) and their implementation in health care (stand-alone intervention, blended care, and stepped and/or collaborative care). Thus, little is known about the effectiveness and acceptance of TBIs concerning their specific application area (eg, as stand-alone interventions for acute depression treatment), as the majority of systematic reviews focus on the assessment of a specific TBI in general, such as computerized CBT (cCBT) for depression [18]. Thus, current guideline recommendations are still limited to the general effectiveness of cCBT [1,7]. Given the large heterogeneity of TBIs, it is of great relevance—especially when deciding on the implementation of TBIs for health care systems—to determine the differential indication of TBIs considering structural (eg, different clinical phases of depression management), interventional (eg, technical medium of intervention delivery), and person-related (eg, symptom severity) determinants. This is the only way to answer what kind of TBIs are effective, accepted, and safe for whom under specific circumstances. Therefore, we aim to build and structure a comprehensive evidence base.

Second, to date, there is only one systematic review evaluating internet- and mobile-based interventions in people with formally diagnosed depression [19]. However, the vast majority of synthesizing research in this field includes studies based on cutoff scores of depression rating scales (ie, focusing on depressive symptoms) rather than focusing on studies using a formal diagnostic process (ie, focusing on depressive disorders), which is in turn required to initiate treatment (and not only prevention) in the mental health care system. In addition, high-quality evidence (RCTs) in clinical samples is the preferred source of evidence for the development and updating of clinical treatment guidelines, such as the German [1] and the United Kingdom [7] guidelines for depression.

Third, there is little research considering different types of negative events with regard to TBIs [21]. Although there are 2 meta-analyses assessing the safety of TBIs, both studies focused only on depressive symptom deterioration in guided [22] and self-administered [23] internet-based therapy. However, other types of negative events, such as treatment dropout, serious adverse events, nonresponse, or unwanted events (eg, frustration caused by technical problems) may occur during the course of internet-based therapy, which is relevant for safety assessment. In addition, depressive symptom deterioration was only assessed for a specific subsample of TBIs; deterioration regarding other delivery modes, such as telephone therapy, is still unknown. However, a comprehensive safety assessment is indispensable for reliable guideline recommendations, patient education, and individual treatment recommendations. By capturing whether (considering different types of) negative events are reported in the included studies, we aim to prestructure evidence for subsequent safety assessments on TBIs.

We chose the methodology of a systematic review to structure a broad and rapidly growing research field. First, the systematic review should provide an overview considering published and unpublished evidence—including gray literature—in the field of TBIs for the treatment of depression. Second, by considering relevant aspects of TBIs as defined by Ebert et al [13], we aim to structure available evidence to build a comprehensive evidence base for a subsequent, more differentiated assessment of effectiveness, acceptance, and safety on TBIs and to identify research gaps.

In summary, our main aim is to structure available evidence on TBIs for the treatment of diagnosed depression, addressing the following research questions:

- How much high-quality evidence (ie, RCTs) on TBIs in the treatment of diagnosed depression is available?
- How is the evidence on TBIs distributed regarding general study characteristics (eg, year of publication)?
- How is the evidence on TBIs distributed regarding treatment characteristics (investigated TBI programs, technologies for intervention delivery, degree and purpose of guidance, qualification of people providing guidance, intervention duration, and therapeutic rationale) and application areas (different clinical phases of depression management and their implementation in mental health care)?
- Are negative events reported in studies of TBIs for the treatment of diagnosed depression and what kind of negative events are addressed (eg, symptom deterioration, nonresponse)?

## Methods

This study was preregistered (PROSPERO registration number CRD42016050413), and a study protocol was published a priori [12]. This review is in accordance with the standards of the Cochrane Collaboration [24] (eg, preregistration, study protocol, systematic literature search considering gray literature, risk of bias assessment, statistical methods of data syntheses) and reported in line with the PRISMA (Preferred Reporting Items for Systematic Reviews and Meta-Analyses) statement [25].

### Inclusion and Exclusion Criteria

We included studies if (1) the whole sample (≥80%) consisted of people (aged ≥18 years) diagnosed with unipolar depression (assessed by a formal classification system or by conducting a diagnostic interview) with any comorbidities and in any clinical phase of depression management, (2) the intervention was at least partly delivered through technical devices (eg, smartphones, computers, telephones), (3) the intervention was based on an explicit psychotherapeutic theory, and (4) the study was conducted as a (cluster) RCT.

We excluded studies if (1) participants were solely diagnosed by applying cutoff scores on depression scales or when they had a depressive episode in the course of a bipolar disorder; (2) concurrent conditions (either somatic or mental) were the main focus of the intervention; and (3) the intervention provided solely psychoeducational content, patient decision aids, depression management tools, or focused only on drug adherence.

The study protocol by Köhnen et al [12] provides more details on definitions and eligibility criteria.

### Search Strategy

We searched the following key databases: CENTRAL (Cochrane Central Register of Controlled Trials), MEDLINE, PsycINFO, PSYNDEX, and CINAHL; see the study protocol by Köhnen et al [12] for the search strategies. The search was not limited by date, language, or publication status. We further searched clinical trial registers (ClinicalTrials.gov, International Clinical Trial Registry Platform, German Clinical Trial Register) and sources of gray literature (Open Grey, Trip Database, ProQuest Dissertations & Theses Abstract and Indexing, and [specialized registers of] ISI Web of Science). Finally, we contacted all the first authors of the included studies for additional information on (un)published trials and supplementary information or the status of ongoing studies (preregistered trials and study protocols).

### Selection Procedure

In total, 2 reviewers (MK and SL) independently screened the first 100 records for inclusion. As the interrater reliability for this sample was found to be high (98%), only one reviewer (MK) screened the remaining records in the course of the title or abstract screening. However, a second reviewer (SL) assessed publications labeled as *unclear*. Selected full-text publications were subsequently assessed for inclusion by 2 independent reviewers (MK and MD). Discrepancies were resolved by discussion with a third reviewer (SL).

### Data Extraction

We developed and piloted a standardized data extraction sheet containing characteristics of interest (see study protocol by Köhnen et al [12] for further information on extracted data). The data extraction sheet comprised information regarding general study information (eg, authors), methodological characteristics (including the risk of bias assessment), sample characteristics (eg, age), treatment characteristics, application areas, sample size and study flow, and primary (posttreatment scores of depression and treatment dropout rates) and secondary (eg, remission rates, quality of life) outcome data.

Essential characteristics were either judged (risk of bias assessment [24], rating of included trials on the efficacy-effectiveness spectrum [26]) or extracted (primary and secondary outcomes) independently by 2 reviewers (MK and MD or TS or Eileen Wehmann). Half of the included studies were extracted completely by 2 independent reviewers; in the other half, further characteristics (eg, therapeutic rationale of TBIs) were extracted by one reviewer (MK). Disagreements were resolved by discussion or by consulting a further reviewer (SL). As the aim was to structure evidence for TBIs, not all extracted data will be reported in this publication.

The risk of bias assessment was evaluated using the criteria described in the Cochrane Handbook for Systematic Reviews of Interventions [24] (including the domains: random sequence generation, allocation concealment, blinding of participants and personnel, blinding of outcome assessment, incomplete outcome data, selective outcome reporting, and other bias). In line with a previous operationalization [27], we specified the domain *other bias* using the following 3 categories: insufficient treatment adherence, allegiance bias, and attention bias.

### Data Extraction: Classification of Negative Events

We applied the recommended definition of negative effects from the *consensus statement on defining and measuring negative effects of internet interventions* [21] to describe if negative effects are reported in included studies. We waived using the term *effect* in this context, as it implies a causal relationship between treatment and harmful outcome; thus, we used the term *event* implying that a harmful outcome has occurred during or after treatment, independent of whether it was caused by it [24].

According to this, negative events comprise the following categories:

- Deterioration: worsening of target symptoms in the course of treatment measured by validated target symptom scales [21].
- Adverse events: any types of adverse events occurring during or after treatment, including physical symptoms (eg, headache); psychological symptoms (eg, depressed mood); and psychosocial, legal, and economic consequences (eg, conflicts with the partner) [27].
- Severe adverse events: any type of adverse events leading to serious consequences, such as death, mortal danger, hospitalization, or disability [28].
- Novel symptoms: novel symptoms describe the emergence of new psychological symptoms (other than the symptoms addressed in treatment), independent of whether novel symptoms are associated with treatment [21].
- Dropout from treatment.
- Nonresponse.
- Unwanted events: any type of events that are experienced as negative by patients in the course of the treatment, independently of whether unwanted events are associated with the treatment being used. In addition, unwanted events are not necessarily related to the treatment outcome, such as technical issues causing frustration or social stigma [21].

### Data Analysis

We structured the included studies according to application areas: clinical phases of depression management consisted of waiting periods, acute treatment, and aftercare. Within different phases of depression management, TBIs can be distinguished concerning their implementation in mental health care. They can be delivered as stand-alone interventions (TBIs replacing face-to-face [F2F] therapy), as blended treatments (combining TBIs and F2F therapies), or as part of stepped (TBIs are used as low threshold, initial treatment options for people with a mild-to-moderate depressive disorder) and/or collaborative care models (TBIs may be provided alongside different treatment components, such as a TBI is offered in addition to a monitoring care manager, general practitioners’ care, and the provision of an online discussion forum). In addition, treatment characteristics were used to structure the available evidence. We used descriptive statistics (eg, frequencies, measures of central tendency, measures of variability) for quantitative analysis using Microsoft Excel 2013 (Microsoft).

### Patient Involvement

We actively involved patients and their relatives in the process of conducting our systematic review by means of 2 workshops (see study protocol by Köhnen et al [12] for details). The first workshop provided general information on systematic reviews and TBIs, and we collected the most relevant outcome domains concerning TBIs from a patient or relative perspective. The second workshop provided study findings and discussed the results of reporting practices concerning these patient-relevant outcomes.

## Results

### Search Results

**Figure 1:**
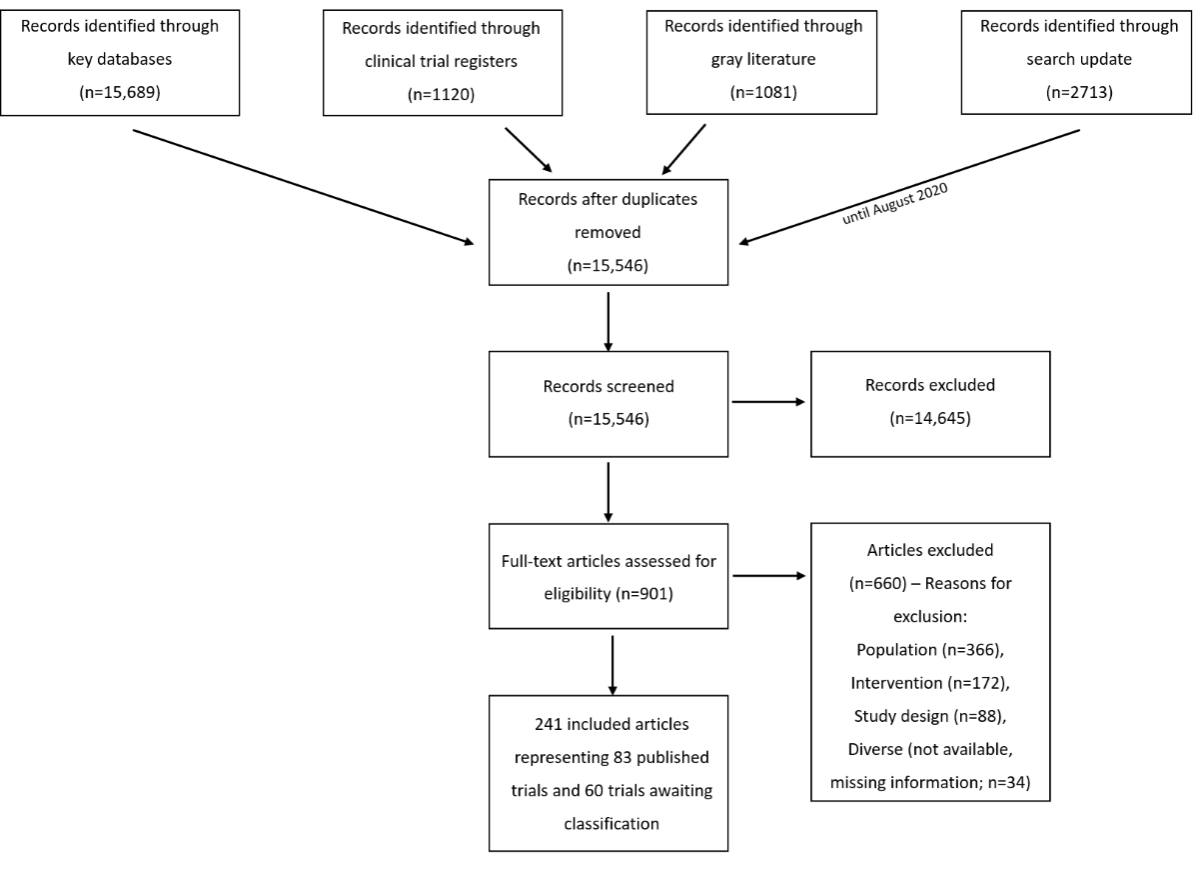
Preferred Reporting Items for Systematic Reviews and Meta-Analyses (PRISMA) flowchart.

Electronic searches yielded 20,613 records. After deduplication, 15,546 records were screened by title or abstract. In total, 901 full-text articles were assessed for eligibility. Not fulfilling the population criteria was the major reason (366/901, 40.6%) for exclusion, as many studies included their participants by applying cutoff scores on depression rating scales, rather than including participants on the basis of a formal diagnostic process (eg, clinical interview). Other reasons for exclusion were not fulfilling intervention (172/901, 19.1%) and study design (88/901, 9.8%) criteria. The remaining studies (34/901, 4.0%) were excluded for diverse reasons: publications were unavailable or relevant study information was missing (eg, distribution of diagnoses across the sample) and also not retrievable by contacting corresponding authors. Overall, we included 241 publications representing 143 trials (83 published trials and 60 trials awaiting classification) covering all clinical phases of depression management. Figure 1 provides a detailed study flow.

### General Study Characteristics

Overall, the identified studies (N=83) included 14,080 participants, ranging from 14 to 1089 per study. The mean age of the participants was 44.9 (SD 12.1) years, and two-thirds were female (67%; see Multimedia Appendix 1 for details [15-17,29-108]).

Most included trials had a trial registration (58/83, 70%), and approximately one-fourth (22/83, 27%) of the included trials had an accompanying study protocol (Table 1). The most common source of risk of bias was nonblinding of participants and personnel (as blinding is barely possible in psychotherapy research), selective reporting, and other bias (especially because of insufficient treatment adherence; see Multimedia Appendix 2 for details)

Studies on TBIs for depression were published from 1990 to the date of our search update in August 2020 (Table 2). The geographical region and country of trials are shown in Table 3.

**Table 1:** General study characteristics (N=83).

Variables			Studies, n (%)
**Registration of studies and publication of study protocols**			
	**Trials with study registration**		58 (70)
		With study protocol	21 (25)
		Without study protocol	37 (45)
	**Trials without study registration**		25 (29)
		With study protocol	1 (1)
		Without study protocol	24 (28)
**Number of study arms in included trials**			
	2		64 (77)
	3		16 (19)
	≥4		3 (4)

**Table 2:** Publications on TBIs for depression per decade (N=83).

Decade	Studies, n (%)
1990 to 1999	2 (2)
2000 to 2009	4 (5)
2010 to 2019	69 (83)
2020 (end of August)	8 (10)

**Table 3:** Trials by geographical region (N=83).

Geographical region	Studies, n (%)
Europe	44 (53)
North America	23 (28)
Australia	9 (11)
Asia	7 (8)

### Treatment Characteristics

#### Investigated TBI Programs

Overall, 26 specific TBI programs were evaluated in the included studies; 18 of these programs were evaluated by 1 study, and 8 were evaluated by more than 1 study. However, approximately half of the studies (40/83, 48%) did not provide a name for the applied TBI program (Multimedia Appendix 3 [15-17,29-109]).

#### Technologies for Intervention Delivery

We identified 107 arms (from 189 arms) in the included studies that applied TBIs. Most TBIs (78%) were delivered by one technical medium (eg, internet or telephone), whereas 22% of TBIs applied more than one technical medium (eg, internet and telephone). Most TBIs were delivered via the internet (54%), followed by telephone (11%), offline computer programs (7%), videoconferencing tools (3%), and mobile phones delivering text messages (2%; see Multimedia Appendix 4 for details).

### Purpose of Guidance

The purpose of guidance in TBIs was heterogeneous (Multimedia Appendix 3).

To structure the guidance in TBIs, we summarized the reported purposes of guidance to categories and identified 5 functions of guidance: (1) informative function (eg, answering queries related to technical issues or treatment), (2) monitoring function (eg, symptom monitoring), (3) adherence-facilitating or motivational function (eg, encouragement to continue with intervention), (4) feedback function (eg, providing feedback for homework), and (5) therapeutic function (eg, goal setting).

Most guided TBIs fulfilled more than one function addressing different needs of participants.

### Degree of Guidance in TBIs

We rated the degree of guidance in TBIs according to the framework of Newman et al [109], consisting of 4 categories, as follows: self-administered therapy, predominantly self-help, minimal-contact therapy, and predominantly therapist-administered intervention.

Trials applying blended treatments were classified in an extra category because these trials provide F2F guidance (eg, by psychotherapists) The included trials applied TBI arms that were either unguided (20/107, 18.7%), guided (59/107, 55.1%; combination of predominantly self-help, 46/107, 43.0%, and minimal contact therapy, 13/107, 12.1%), therapist-delivered (14/107, 13.1%), or blended treatments (14/107, 13.1%).

### Qualifications of People Providing Guidance

The qualification of people who provided guidance on TBIs and who delivered treatment via TBIs ranged from lay supporters (technicians, research assistants, etc; 8/71, 11%) to clinicians with experience in the treatment of people with mental illness (trained psychotherapists, 6/71, 8% as well as psychiatrists, 1/71, 1%). Most people providing guidance and delivered treatment via TBIs had a background in psychology (36/71, 51%).

### Interventions’ Duration

Interventions’ duration of identified TBIs ranged from 1 to 52 weeks, with most interventions lasting between 6 and 12 weeks (median treatment length of 8.5 weeks). Interventions of 8-week duration were the most frequent (26/107, 24.3%) in the included studies.

### Therapeutic Rationale of TBIs

Overall, we identified 15 different therapeutic rationales for TBIs, ranging from mindfulness to psychodynamic therapy. Most TBIs were based on cognitive behavioral treatment approaches (79%; Multimedia Appendix 5).

### Application Areas of TBIs

**Figure 2:**
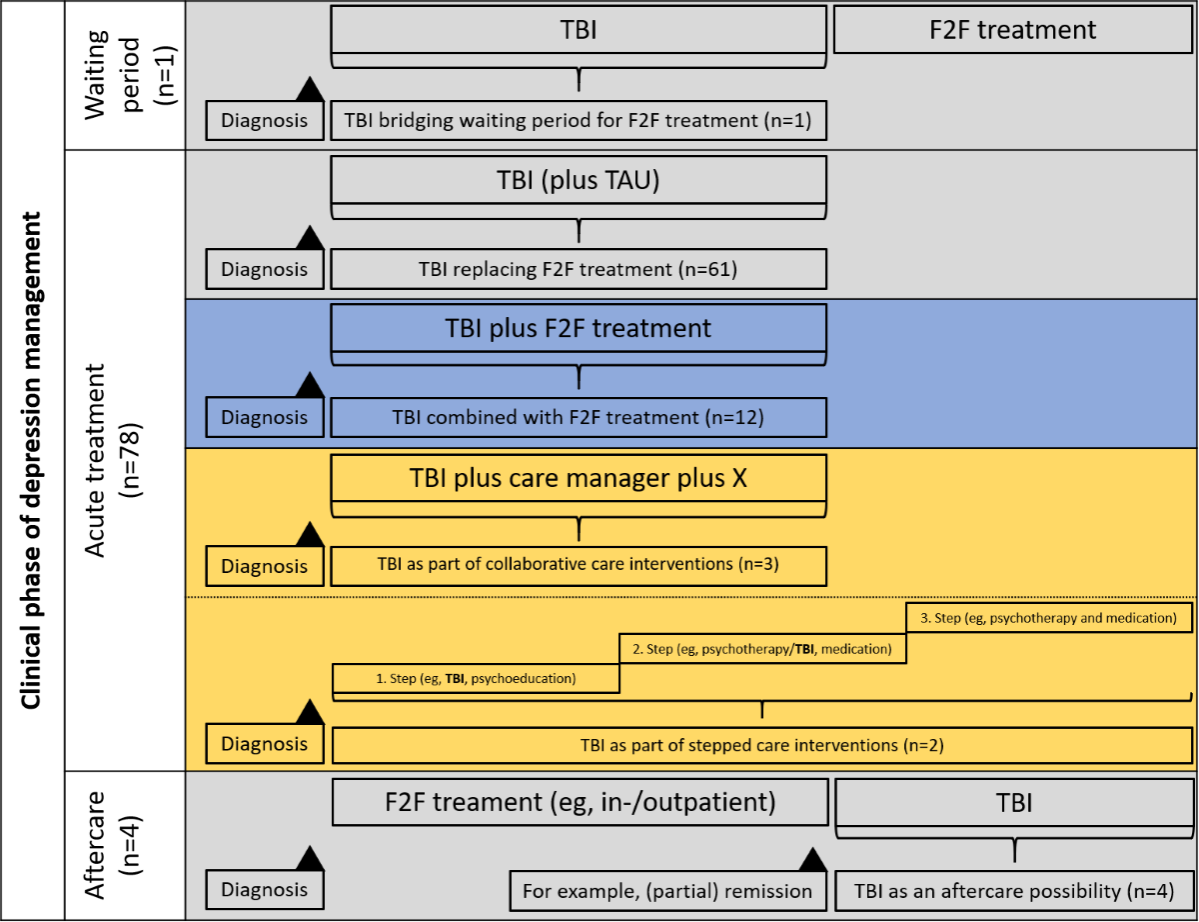
Distribution of studies (N=83) on application areas. Color highlighting of cells indicates format of implementation of TBIs: grey = (enhanced) stand-alone intervention; blue = blended treatment approach; yellow = TBI as part of collaborative/stepped care interventions. TAU: treatment as usual; TBI: technology-based psychological intervention; F2F: face-to-face.

Concerning the clinical phase of depression management, almost all trials were related to the acute treatment of people with acute depression (94%), followed by trials addressing the aftercare of people with depression (5%), and one trial that applied a TBI as a tool for bridging waiting periods (1%). Regarding the implementation of mental health care, most TBIs were delivered as a (enhanced) stand-alone intervention (80%), followed by blended treatment approaches (14%), and 5 studies (6%) delivered TBIs as part of a collaborative (4%) or stepped (2%) care interventions (Figure 2).

We applied a rather broad definition for blended treatments, since we included all studies that provided any type of F2F treatment tailored to depression (eg, psychotherapy, medication, depression specific GP care) in addition to TBIs irrespective of the study’s definition/label. In contrast, trials concurrently providing treatment as usual in addition to TBIs were considered as enhanced stand-alone interventions, if treatment as usual consisted of a systematically offered generic treatments (eg, general GP care for all participants) that was not specifically tailored to depression.

### Report of Negative Events

Most studies (70/83, 84%) reported dropout rates. However, reporting on dropouts is heterogeneous, as studies differed in their definitions on dropouts: there were studies reporting dropouts from treatment (or treatment completion rates; 38/83, 46%), whereas other studies applied other definitions of dropouts, for instance, treatment completers as defined by authors or withdrawals from the study (32/83, 39%), and approximately 16% (13/83) of included studies did not report any extractable dropouts (Table 4; see Multimedia Appendix 6 [15-17,29-108] for an overview on negative events). On average, the included trials reported on 1.67 (SD 1.06; range 0-5) categories of negative events (out of 7 potential categories).

**Table 4:** Report of negative events in included studies (N=83).

Report of negative events	Studies, n (%)
Dropout	70 (84)
Deterioration	21 (25)
Adverse events	18 (22)
Nonresponse	16 (19)
Severe adverse events	14 (17)
Novel Symptoms	0 (0)
Unwanted Events	0 (0)

## Discussion

### Principal Findings

The aim of our study was to structure available evidence on TBIs for the treatment of diagnosed depression to build a comprehensive evidence base and to identify research gaps.

### Application Areas

As shown in Figure 2, the vast majority (94%) of the included studies focused on the acute treatment phase. Significantly less evidence was available for TBIs in aftercare (5%) and for TBIs bridging waiting periods (1%), indicating research gaps despite extensive discussions on their usefulness in these clinical phases of depression management. For example, TBIs for bridging waiting periods may help to establish early symptom reduction [11] or to counteract symptom manifestation, which may prevent aggravation, recurrence, and the experience of a persistent course for people on waiting lists for treatment. Furthermore, TBIs may prepare for F2F treatments by providing, for example, psychoeducational information on depression (eg, symptoms) [11] so that there is subsequently more time for working on therapeutic content (eg, behavioral activation). TBIs in aftercare are seen as (potentially) useful tools providing an aftercare possibility attached to inpatient treatment with lower barriers (eg, waiting periods) compared with traditional aftercare approaches [110]. In view of the fact that we have identified only a few studies for aftercare [16,29-31] and one for bridging waiting periods [32], the question arises whether firm conclusions about the effectiveness and acceptance of these clinical phases can be made on the basis of the available evidence. This is also supported by looking at the level of synthesizing research, as only 2 reviews [111,112], which dealt with one of these phases, could be identified. However, even if these reviews were based on broader inclusion criteria (eg, the presence of a [former] depressive disorder was not required), they could identify only a few—quite heterogeneous—studies and could not draw firm conclusions about effectiveness and acceptance in these clinical phases of depression care [111,112]. Given the unconvincing evidence base and the probable potential of TBIs to overcome treatment barriers for aftercare and in bridging waiting periods, it is of great relevance to conduct research on TBIs in these specific clinical phases of depression management, at best pragmatic large-scale RCTs with people having diagnosed depression, so that a comprehensive assessment, also decisive for guideline recommendations, is possible in the future.

In addition, most studies implemented TBIs either as (enhanced) stand-alone interventions (80%) or as blended treatment approaches (14%) in the acute treatment phase of depression treatment, indicating a comprehensive evidence base useful for further analyses on the differential indication for this clinical phase of depression management. There is little evidence on TBIs as part of collaborative (4%) or stepped (2%) care interventions in our review (Figure 2), which may be traced back to the fact that studies were only considered if the results were differentially reported for the technology-based treatment component.

Stepped care approaches incorporating TBIs are recommended by the German [1] and the United Kingdom [7] guidelines for improving depression care. In addition, stepped care approaches are seen as promising options to up-scale (depression) treatment options being concurrently more cost-effective compared with other approaches, especially when TBIs are integrated. Thus, care within stepped care models is initially offered as a low-threshold (and low-cost) intervention with constant symptom monitoring. When patients do not respond to an intervention, they will be stepped up receiving more intensive interventions. However, to the best of our knowledge, there is no meta-analysis on the effectiveness of stepped care approaches with TBIs, indicating that a sufficient evidence base is missing. In contrast, traditional stepped care approaches without TBIs have been found to be effective for treating depression [113]. Thus, not surprisingly, we only identified 2 studies [33,34] offering TBIs (internet-based CBT) as a low-threshold intervention in the course of a stepped care approach. To assess the usefulness of specific TBIs within stepped care approaches, we need studies testing different treatment options comparatively at different levels of the stepped care approach (for instance, first step: watchful waiting vs iCBT (internet-based CBT) vs bibliotherapy; second step: F2F psychotherapy vs telephone psychotherapy). In addition, studies would be useful for assessing both the whole stepped care approach depending on whether a TBI component was implemented (eg, [non]provision of iCBT as a low-threshold first-step intervention) and the benefit of these components within the stepped care approach (eg, pre-post gains, stepping-up rates, step-specific adherence rates).

In summary, there is evidence on the acute treatment phase of depression, and there are promising approaches to improve mental health care for people with depression by using TBIs. However, there are only a few studies investigating TBIs outside of acute treatment and applying innovative treatment approaches, which is why we call for (1) more research in previously less-considered clinical phases of depression management (aftercare, waiting periods) and (2) more studies investigating stepped care approaches with different TBI components.

### Treatment Characteristics

We found that most TBIs were based on cognitive behavioral treatment approaches (79%), especially CBT (65%), and that other guideline-recommended treatment rationales for F2F depression treatment, such as psychodynamic treatments, are barely researched in TBIs of diagnosed depression. This may be because of the fact that psychodynamic-oriented therapists have a more negative attitude toward internet interventions compared with other therapists [114]. In addition, there is an ongoing debate in the psychodynamic community—at least in German-speaking countries—concerning whether an adequate therapeutic alliance, which is emphasized being a central treatment component, can be established in treatments outside of the F2F setting, as, for example, certain cues (visual and/or auditive) are missing [115]. However, recent reviews suggest that establishing a sustainable therapeutic alliance may be possible when treatment is delivered by different technical mediums [116,117]. Another reason cognitive behavioral rationales are particularly suitable may be because of their more manualized content [118]. Content intended for F2F treatment may be easier transferred to other settings and, for example, made available via web-based programs.

Owing to the lack of studies investigating other approaches recommended by guidelines, we call for more studies on TBIs considering a broader variety of treatment approaches. This would allow for more differentiated guideline recommendations, as they are currently limited to the effectiveness of cCBT [1,7]. In addition, patient preferences regarding TBIs could be considered when treatment is planned because preferences (CBT vs psychodynamic therapy) seem to have predictive value for treatment outcome in internet-delivered interventions [119] and significantly affect outcomes in regular treatment of mental disorders [120].

### Report of Negative Events

Considering the report of negative events in included studies, we found that apart from dropouts, other negative events such as deterioration, nonresponse, and (severe) adverse events were reported in a few studies (range 17%-25%) or not at all (unwanted events and novel symptoms). Although dropouts were reported by most studies (84%), there were reported quite heterogeneously, as only 46% of all studies reported dropouts from treatment (or completion rates), which is an important indicator for treatment adherence in TBIs. For example, it is well known that unguided TBIs produce significantly more dropouts than guided TBIs [18]. Dropout rates (as an indicator for treatment adherence and therefore also for safety) have to be considered when comparing other treatment characteristics (eg, video vs telephone). Moreover, 39% of studies reported other kinds of dropouts (eg, withdrawals from study, treatment completers, as defined by the authors). This kind of dropouts is less meaningful, as the link to treatment adherence or safety is less clear.

Although adverse events have been reported in 22% of studies, many studies have reported adverse events unsystematically. For instance, by stating that no adverse events have been noted for any study participants, which did not specify the method of capturing adverse events as well as the definition of adverse events in included trials. It was not clear if participants were asked about the occurrence of adverse events during or after treatment. On the other hand, there were trials systematically assessing adverse events, for instance, by mapping them to different symptom domains (eg, somatically and psychologically) and specifying time points for the assessment. In total, the method on how and when adverse events were captured remained unclear in most included studies, which may contribute to an underestimation of the occurrence of adverse events because it is more likely to report such events when specifically asked for it in comparison to spontaneous reports [121,122].

Our findings on the report of negative events are in line with a previous systematic review, which also noted that adverse events were heterogeneous and insufficiently reported in RCTs on people with a persistent depressive disorder, especially in psychotherapeutic studies, where the report of adverse events was largely neglected [123]. However, in our review, when all categories of negative events were considered, almost all studies reported at least some information on negative events (93%); nonetheless, the reporting between studies on certain categories (eg, dropouts) was inconsistent. This inconsistency may be explained by the fact that included trials considered more generic reporting guidelines (eg, Consolidated Standards of Reporting Trials [CONSORT]), rather than considering specific guidelines or guideline extensions (eg, CONSORT Extensions [for harms]), as they are rarely endorsed by high-impact journals [124], which may also influence authors’ use [125].

Given the great heterogeneity in the reporting of negative events in included studies, we suggest the use of specific guidelines or guideline extensions to future trialists, such as the CONSORT extensions for harms [126], which may help make the report more precise and homogeneous, by for instance clarifying how information concerning negative events was collected.

### Strengths and Limitations

Our review was conducted in line with the Cochrane guidelines, reported following PRISMA guidelines [25], and studies were selected according to prespecified criteria [12], reflecting high methodological standards. However, we deviated from the study protocol by waiving an additional forward and backward reference search because our systematic literature search was already very comprehensive.

Despite strict eligibility criteria (eg, diagnosed depression), the focus of our review is still broad because all TBIs were considered irrespective of application areas and certain treatment characteristics. Following a broad focus resulted in large heterogeneity of the included studies, which is probably challenging for subsequent meta-analyses. It may be possible that certain questions regarding the differential indication are unsuitable for evidence synthesis because of large heterogeneity (eg, differences in intervention duration) or because there are not enough studies available (eg, evidence for TBIs bridging waiting periods [n=1]).

We conducted a highly sensitive literature search considering key databases, databases of gray literature, and clinical trial registries, without limiting the literature search to language. Nonetheless, we may have missed trials published in languages other than English because databases containing primarily English records may fail to find trials published in other languages even when language restrictions were avoided [127].

### Conclusions

Overall, the results indicated that there is a proper evidence base for TBIs in the acute treatment phase being either implemented as stand-alone or blended treatments. However, the evidence base of TBIs in aftercare or for bridging waiting periods was found to be hardly convincing. Moreover, most TBIs were theoretically based on cognitive behavioral treatment rationales. Thus, a (broader) evidence base including TBIs based on other therapeutic rationales is still missing.

Concerning the report of negative events in studies evaluating TBIs, it was found that some information on negative events was reported in almost all studies, but the report was quite inconsistent between studies.

Despite the unequal distribution of evidence concerning differing clinical phases of depression management and treatment characteristics, we compiled a comprehensive evidence base to subsequently assess the effectiveness, safety, and acceptance of TBIs.

## Appendix

Multimedia Appendix 1 Study characteristics.

Multimedia Appendix 2 Risk of bias assessment.

Multimedia Appendix 3 Treatment characteristics of technology-based psychological interventions.

Multimedia Appendix 4 Technologies for intervention delivery.

Multimedia Appendix 5 Therapeutic rationales of technology-based psychological interventions.

Multimedia Appendix 6 Report of negative events.

## Abbreviations

CBT: cognitive behavioral therapy
cCBT: computerized cognitive behavioral therapy
CENTRAL: Cochrane Central Register of Controlled Trials
CONSORT: Consolidated Standards of Reporting Trials
F2F: face-to-face
iCBT: internet-based CBT
PRISMA: Preferred Reporting Items for Systematic Reviews and Meta-Analyses
RCT: randomized controlled trial
TBI: technology-based psychological intervention
