# Supplementary material for: Evidence on Technology-Based Psychological Interventions in Diagnosed Depression: Systematic Review
Source: JMIR Ment Health. 2021 Feb 10;8(2):e21700. doi: 10.2196/21700 (PMC7904404; doi:10.2196/21700)
Supplement: Multimedia Appendix 1 [file mental_v8i2e21700_app1.pdf]

Appendix 1. Study characteristics.

Study ID (Author / year / reference)	Location of study	N	Study arms	Trial registration / study protocol	Recruitment	Depression Diagnosis	Clinical phase of depress-ion management	Implementat ion in mental health care	Age – M (SD)	Sex (% of females)	Primary outcome applicability for meta-analysis	
											PT-Depres sion scores (yes / no)	PT-Drop-out rates (yes / no)
Agyapong (2017) [35]	Canada	73	1) Supportive text messages<br>2) control text messages	no / no	Secondary care (recruited via four community mental health care clinics)	MDD	Acute treatment	Stand-alone intervention	ND	68.5	yes	yes
Andersson (2013) [36]	Sweden	69	1) Internet-based cognitive behavioral therapy<br>2) in-person group cognitive behavioral therapy	no / no	Community (advertisements)	MDD (71.0% in recurrent episode, 1.45% in single episode, Dysthymia (6.0%), MDD plus dysthymia 21.7%)	Acute treatment	Stand-alone intervention	42.3 (13.5)	78.3	yes	yes
Arjadi (2018) [37]	Indonesi a	313	1) Internet-based behavioral activation intervention<br>2) Online psychoeducation	yes / yes	Community (advertisements) & referrals from institutions or mental health professionals	MDD (mild (24.16%), moderate (31.19%), severe 40.89%, based on PHQ-9)	Acute treatment	Stand-alone intervention	ND	80.8	yes	yes

Berger (2011) [38]	Switzerland, Germany	76	1) Guided internet-based CBT<br>2) unguided internet-based CBT<br>3) waiting list	no / no	Community (advertisements)	MDD, Dysthymia (9.2%); MDD severity (mild (22.4%), moderate (60.5%), severe (7.9%), based on BDI-II scores)	Acute treatment	Stand-alone intervention	38.8 (14.0)	69.7	yes	yes
Berger (2018) [39]	Germany	98	1) regular f2f psychotherapy plus internet-based CBT (Deprexis)<br>2) regular f2f psychotherapy	yes / yes	Secondary care (outpatient psychotherapeutic practices)	(ICD-10; F32.-Depressive Episode, F33.-Recurrent Depressive Disorder, F34.-Persistent Affective Disorder, F38.-Other Affective Disorder, F39 Unspecified Affective Disorder)	Acute treatment	Blended treatment	43.1 (12.0)	66.3	yes	no
Blackwell (2015) [40]	UK	150	1) imagery cognitive bias modification<br>2) control condition (without training contingency, which was provided for other intervention arm)	yes / no	Community (advertisements)	MDD	Acute treatment	Stand-alone intervention	ND	68.7	yes	yes
Bowers (1993) [41]	USA	22	1) TAU<br>2) TAU plus therapist delivered CBT	no / no	Secondary care (inpatient psychiatry service)	MDD	Acute treatment	Blended treatment	ND	68.2	yes	no

			3) TAU plus computer assisted CBT									
Carlbring (2013) [42]	Sweden	80	1) Computer-administered therapy<br>2) waiting list	no / no	Community (advertisement)	MDD	Acute treatment	Stand-alone intervention	44.50 (13.50)	82.5	yes	no
Celano (2017) [43]	USA	65	1) Positive psychology<br>2) cognition focused condition	yes / no	Secondary care (inpatient psychiatric units)	MDD	Acute treatment	Enhanced stand-alone intervention	ND	69.2	yes	no
Choi (2012) [44]	Australia	63	1) Internet-based CBT (attuned to Chinese people with depression living in Australia)<br>2) waiting list	yes / no	Community (online recruitment)	MDD	Acute treatment	Stand-alone intervention	39.0 (11.7)	80	yes	no
Choi (2014) [45]	USA	158	1) Videoconferencing problem solving therapy, 2) face-to-face problem solving therapy<br>3) telephone support calls	no / no	Recruited by case managers	MDD (65.2%), depressive disorder, NOS (29.7%), dysthymia (5.1%)	Acute treatment	Stand-alone intervention	64.80 (9.18)	78.5	yes	yes
Corruble (2016) [46]	France	221	1) Social rhythm therapy plus face-to-face treatment with a psychiatrist and antidepressant medication<br>2) clinical management plus face-to-face treatment with a	yes / no	Secondary care (psychiatrists of outpatients)	MDD	Acute treatment	Blended treatment	ND	ND	no	no

			psychiatrist and antidepressant medication<br>3) TAU (posteriori matched control group)								
Egede (2015) [47]	USA	241	1) Videoconferencing behavioral activation treatment<br>2) face-to-face behavioral activation treatment	yes / yes	Institutional (veterans affairs medical center), secondary care (outpatient based clinics)	MDD	Acute treatment	Stand-alone intervention	63.90 (5.10)	2	no	yes
Forand (2018) [48]	USA	90	1) Internet-based CBT<br>2) waiting list	yes / no	Community (advertisements)	MDD (98%)	Acute treatment	Stand-alone intervention	ND	75	yes	no
Forsell (2017) [49]	Sweden	42	1) Internet-based CBT plus antenatal TAU<br>2) antenatal TAU	yes / no	Community (advertisements)	MDD	Acute treatment	Enhanced stand-alone intervention	ND	100	yes	no
Gilbody (2016) [50]	UK	691	1) Internet-based CBT (Beating the Blues) plus usual GP care<br>2) internet-based CBT (MoodGYM) plus usual GP care)<br>3) usual GP care	yes / yes	Primary care (general practices)	MDD (81%)	Acute treatment	Enhanced stand-alone intervention	39.86 (12.65)	67	yes	no
Graaf (2009) [51]	Netherlands	303	1) Internet-based CBT plus TAU<br>2) internet-based CBT<br>3) TAU	yes / yes	Community (advertisement, invitation)	Recurrent MDD (36.30%), single MDD (43.56%)	Acute treatment	1) Enhanced stand-alone intervention<br>2) stand-alone intervention	ND	56.8	yes	no

Holländare (2011) [16]	Sweden	84	1) Internet-based CBT<br>2) possibility of non-specific e-mail support	no / no	Community (advertisements)	MDD (100% in partial remission with no current episode of MDD at the time of recruitment)	Aftercare	Stand-alone intervention	45.3 (12.8)	84.5	yes	yes
Hunkeler (2012) [52]	USA	103	1) Collaborative care website plus usual specialty mental health care<br>2) usual specialty mental health care	no / no	Secondary care (mental health care clinics)	Recurrent MDD	Acute treatment	Collaborative care approach	ND	79.6	yes	no
Johansson (2012) [53]	Sweden	92	1) Internet-based psychodynamic treatment<br>2) structured support control group	yes / no	Community (advertisement) and recruitment from a waiting list of another trial	Acute MDD (65.2%), partial remitted MDD (34.8%)	Acute treatment	Stand-alone intervention	45.60 (14.0)	75	yes	no
Johansson (2012) [54]	Sweden	121	1) Tailored internet-based CBT<br>2) standardized internet-based CBT<br>3) participation in online discussion group during waiting period	yes / no	Community (advertisement) and recruitment from online waiting list of people being interested in iCBT	Acute MDD (73.6%), partial remitted MDD (26.4%), dysthymia (14.0%)	Acute treatment	Stand-alone intervention	44.70 (12.10)	71.1	yes	no
Johansson (2013) [55]	Sweden	100	1) Internet-based psychodynamic treatment<br>2) waiting list plus continuous contact with therapist	yes / no	Community (advertisement)	Depression and anxiety disorders, data are reported separately for the depressed subgroup (MDD)	Acute treatment	Stand-alone intervention	ND	ND	yes	no

Kenter (2016) [32]	Netherlands	269	1) Internet-based problem solving therapy<br>2) waitlist plus self-help book	yes / yes	Secondary care (outpatient clinics)	MDD	Bridging waiting period	Stand-alone intervention	38.0 (11.4)	53.9	yes	no
Kessler (2009) [56]	UK	297	1) Online CBT plus usual GP care<br>2) usual GP care while on waitlist	yes / no	Primary care (general practices)	MDD (mild (5.1%), moderate (26.26%), severe (68.69%), based on BDI score)	Acute treatment	Enhanced stand-alone intervention	ND	68.0	yes	no
Kivi (2014) [57]	Sweden	92	1) Internet-based CBT<br>2) TAU	no / no	Primary care (via general practitioners and nurses at primary care centers)	MDD (mild (19%), moderate (34%), severe (41%), based on BDI-II cut-off scores)	Acute treatment	Stand-alone intervention	36.6 (11.3)	66	yes	no
Kok (2015) [29]	Netherlands	239	1) Cognitive therapy plus Mood Monitoring plus TAU<br>2) TAU	yes / yes	Community (via media), primary care (general practitioners), and mental health care institutions	recurrent MDD (with remission/recovery for at least 2 months; mild (23.43%), moderate (53.14%), severe (22.59%), based on the number of SCID-I depression symptoms))	Aftercare	Enhanced stand-alone intervention	ND	74.9	yes	yes
Hirsch (2018) [58]	UK	79	1) Online Cognitive Bias Modification with or, 2) without priming of repetitive negative thinking<br>3) Neutral task control condition	no / yes	Community (via advertisements)	GAD or MDD (outcome data was reported separately)	Acute treatment	Stand-alone intervention	ND	78.5	yes	no

Lam (2013) [59]	Canada	105	1) Telephone CBT plus medication, 2) medication plus adherence reminder	yes / no	Community (advertisement), primary and / or secondary care (referrals)	Single MDD (33.33%), recurrent MDD (66.66%)	Acute treatment	Blended treatment	ND	54.6	yes	yes
Lang (2012) [60]	UK	28	1) Imagery-focused cognitive Bias modification, 2) Control condition with same stimulus material, but with modified contingencies	no / no	Community (advertisement), primary care (local clinicians)	MDD	Acute treatment	Stand-alone intervention	ND	76.9	yes	no
Lappalainen (2015) [61]	Finland	39	1) Internet-based acceptance and commitment therapy<br>2) waiting list	no / no	Community (advertisements)	MDD (mild (25.6%), moderate (43.6%), severe (17.9%), based on BDI-II scores)	Acute treatment	Stand-alone intervention	51.89 (12.88)	71.8	yes	no
Lindner (2014) [62]	Sweden	38	1) Self-help program plus telephone support by therapist<br>2) Self-help program plus e-mail support by therapist	no / no	Community (from waiting list of previous study which was recruited from the general public)	MDD (84.2%), MDD + Dysthymia (15.8%)	Acute treatment	Stand-alone intervention	ND	84.2	yes	yes
Löbner (2018) [63]	German y	647	1) Online CBT plus TAU<br>2) TAU	yes / no	Primary care (general practices)	MDD	Acute treatment	Enhanced stand-alone intervention	ND	68.5	no	no
Luxton (2016) [64]	USA	121	1) In Home treatment<br>2) in-person treatment	yes / yes	Institutional (via military treatment facilities)	MDD (9% suffered from minor depression)	Acute treatment	Stand-alone intervention	ND	18.2	yes	yes

Ly (2014) [65]	Sweden	84	1) Smartphone-based BA intervention<br>2) smartphone-based mindfulness intervention	yes / no	Community (advertisement, mass media)	MDD, MDD and Dysthymia (49%)	Acute treatment	Stand-alone intervention	36.10 (10.80)	70	yes	yes
Ly (2015) [66]	Sweden	93	1) Blended treatment (face-to-session plus smartphone application that were used between the sessions)<br>2) Face-to-face BA	yes / no	Community (advertisement)	MDD	Acute treatment	Blended treatment	30.60 (11.40)	69.9	yes	yes
Mantani (2017) [67]	Japan	164	1) Smartphone CBT plus medication change<br>2) medication change alone	yes / yes	Secondary care (psychiatrists)	MDD	Acute treatment	Blended treatment	ND	53.1	yes	yes
Meyer (2015) [68]	German y	163	1) Internet-based CBT plus permission of using TAU, 2) TAU	yes / no	Recruitment from another trial (, as depressive symptoms were beyond mild to moderate)	MDD (37.2%), Dysthymia (10.4%), MDD and Dysthymia (39.3%), Neither MDD nor Dysthymia (12.9%)	Acute treatment	Enhanced stand-alone intervention	42.0 (11.39)	74.8	yes	no
Milgrom (2016) [69]	Australia	43	1) Internet-based CBT<br>2) TAU	yes / no	Community (advertisements)	Recurrent MDD (67.44%), single MDD (32.26%)	Acute treatment	Stand-alone intervention	ND	100	yes	no
Mohr (2011) [70]	USA	85	1) Telephone CBT<br>2) TAU	yes / no	Secondary care (community-based outpatient clinics)	MDD	Acute treatment	Stand-alone intervention	55.90 (10.59)	9.4	yes	no

Mohr (2012) [71]	USA	325	1) Face-to-face CBT<br>2) Telephone CBT	yes / no	Primary care (primary care clinics), secondary care (general internal medicine clinics)	MDD	Acute treatment	Stand-alone intervention	ND	77.5	no	yes
Mohr (2013) [72]	USA	102	1) Guided internet-based CBT<br>2) unguided internet-based CBT<br>3) waiting list	yes / yes	Secondary care (advertisements in general internal medicine clinic)	MDD	Acute treatment	Enhanced stand-alone intervention	ND	71.4	no	no
Montero-Marin (2016) [73]	Spain	296	1) Self-guided online program plus improved TAU<br>2) therapist-guided online program plus improved TAU<br>3) improved TAU	yes / yes	Primary care (general practitioners)	MDD	Acute treatment	Blended treatment	43.19 (9.3)	79.2	yes	no
Nakao (2018) [74]	Japan	40	1) Blended CBT plus continuation with TAU with their treating psychiatrist<br>2) waiting list and continuation with TAU with their treating psychiatrist	yes / no	Primary care (general hospital), secondary care (teaching and psychiatric hospital)	MDD	Acute treatment	Blended treatment	40.20 (9.70)	50	yes	no
Nyström (2017) [75]	Sweden	312	1) Physical activity without rationale<br>2) physical activity with rationale, 3) BA based on Lewinsohn's model	yes / yes	Community (advertisement)	MDD	Acute treatment	Stand-alone intervention	42.0 (13.5)	76	yes	yes

			4) BA based on Martell's model, 5) waiting list									
O'Mahen (2014) [76]	UK	83	1) BA<br>2) TAU	no / no	Community (advertisements)	MDD	Acute treatment	Stand-alone intervention	ND	100	yes	no
Perini (2009) [17]	Australia	48	1) Online CBT<br>2) waiting list	yes / no	Community (website)	MDD	Acute treatment	Stand-alone intervention	49.29 (12.06)	77.8	yes	no
Reins (2019) [77]	German y	131	1) Internet-based CBT<br>2) online psychoeducation	yes / yes	Community (advertisement)	MDD	Acute treatment	Stand-alone intervention	41.60 (10.80)	75.6	yes	yes
Ren (2016) [78]	China	62	1) Internet-based CBT<br>2) waiting list	no / no	Institutional (via colleges / universities)	MDD	Acute treatment	Stand-alone intervention	ND	ND	yes	yes
Richards (2013) [79]	UK	581	1) Collaborative care intervention plus TAU<br>2) TAU	yes / no	Primary care (general practices)	MDD (mild (14.3%), moderate (55.6%), severe (29.9%), based on ICD-10 diagnosis)	Acute treatment	Collaborative care approach	44.80 (13.30)	71.9	yes	no
Rollman (2018) [80]	USA	704	1) Internet-based CBT supported by collaborative care manager<br>2) Internet-based CBT supported by collaborative care manager plus access to internet support group<br>3) TAU	yes / yes	Primary care (primary care offices)	MDD (81.2%), generalized anxiety disorder (53.5%), panic disorder (15.8%), MDD and anxiety disorder (72.3%)	Acute treatment	Collaborative care approach	42.70 (14.30)	79.8	no	no
Rosso (2017) [81]	USA	77	1) Internet-based CBT<br>2) monitored attention control	yes / no	Community (advertisement)	Recurrent MDD (74.02%), single MDD (25.97%)	Acute treatment	Stand-alone intervention	ND	68.8	yes	yes

Sandoval (2017) [82]	USA	45	1) Interactive media-based, computer-delivered depression treatment program 2) no treatment control condition	no / no	Community (announcements)	MDD (95%), dysthymia (5%)	Acute treatment	Stand-alone intervention	ND	62.22	yes	no
Schlicker (2018) [30]	German y	226	1) Standardized text message intervention 2) individualized text messages 3) waiting control	yes / no	Secondary care (mental health hospital)	MDD	Aftercare	Stand-alone intervention	ND	64.2	yes	yes
Schuver (2016) [83]	USA	40	1) Yoga intervention 2) walking control	yes / no	Community (advertisement)	MDD	Acute treatment	Stand-alone intervention	42.68 (4.95)	100	yes	no
Selmi (1990) [84]	USA	36	1) Computer-administered therapy 2) therapist-administered therapy 3) waiting list	no / no	Community (announcement)	recurrent MDD (19.44%), single MDD (80,56)	Acute treatment	Stand-alone intervention	28.2 (4.58)	63.9	yes	no
Smith (2017) [85]	Australia	270	1) Internet-based CBT, 2) self-help book (CBT), 3) self-help book (meditation), 4) waiting list	yes / no	Community (advertisement) and via virtualclinic.org.a u	MDD	Acute treatment	Stand-alone intervention	ND	81.9	yes	no

Steinmann (2020) [15]	German y	59	1) Telephone CBT with reminding letters<br>2) telephone CBT without reminding letters	yes / no	Primary care (general practitioner)	MDD	Acute treatment	Stand-alone intervention	45.50 (13.60)	72	yes	yes
Thase (2018) [86]	USA	154	1) Blended CBT treatment<br>2) face-to-face CBT	no / no	Community (advertisements) and institutional (clinical services of participating sites)	MDD	Acute treatment	Blended treatment	46.30 (14.30)	66.2	yes	yes
Titov (2010) [87]	Australia	141	1) Internet-based CBT plus technician support<br>2) Internet-based CBT plus clinician support<br>3) waiting list	yes / no	virtualclinic.org.a u	MDD	Acute treatment	Stand-alone intervention	43.00 (12.86)	74	yes	no
Titov (2011) [88]	Australia	77	1) Internet-based CBT<br>2) waiting list	yes / no	virtualclinic.org.a u	MDD (51%) (results for depression subgroup reported separately)	Acute treatment	Stand-alone intervention	43.9 (14.6)	73	yes	no
Torkan (2014) [89]	Iran	39	1) Positive imagery cognitive bias modification, 2) non-imagery control condition	no / no	Secondary care (outpatient clinics)	MDD	Acute treatment	Stand-alone intervention	ND	64.1	yes	no

Vernmark (2010) [90]	Sweden	88	1) Email therapy, 2) internet-based, CBT<br>3) waiting list	no / no	Community (advertisement)	MDD	Acute treatment	Stand-alone intervention	36.82 (12.90)	68.2	yes	no
Watkins (2012) [91]	UK	121	1) Cognitive bias modification plus TAU<br>2) relaxation training plus TAU<br>3) TAU	yes / no	Primary care (general practitioners)	MDD	Acute treatment	Enhanced stand-alone intervention	ND	71.9	yes	no
Watts (2013) [92]	Australia	52	1) Mobile-based intervention (via an application)<br>2) computer-based intervention	yes / no	virtualclinic.org.au	MDD (mild (50%), moderate (23.53%), severe (14.71%), based on PHQ-9 Severity Status)	Acute treatment	Stand-alone intervention	41.00 (12.38)	80	yes	yes
Williams (2013) [93]	Australia	69	1) imagery-focused cognitive bias modification plus internet-based CBT<br>2) waiting list	yes / no	virtualclinic.org.au	MDD	Acute treatment	Stand-alone intervention	ND	76.2	yes	yes

Williams (2015) [94]	Australia	121	1) Positive imagery cognitive bias modification plus internet-based CBT 2) control cognitive bias modification condition plus internet-based CBT	yes / yes	Institutional (not-for-profit clinical and research unit)	MDD	Acute treatment	Stand-alone intervention	ND	73.3	yes	no
Wright (2005) [95]	USA	45	1) Blended cognitive therapy 2) face-to-face cognitive therapy, 3) waiting list	no / no	Community (advertisement), primary and/or secondary care (referral)	single MDD	Acute treatment	Blended treatment	ND	75.6	yes	no
Zagorscak (2018) [96]	German y	1089	1) Internet-based CBT plus standardized, automated feedback and contact on demand 2) internet-based CBT plus semistandardized e-mail feedback	yes / no	Institutional (health insurance)	Single MDD (42.1%), remitted MDD (26.2%), dysthymia (8.3%), double depression (5.3%), Bipolar or NOS (6.0%), no current/past affective disorder (12.2%)	Acute treatment	Stand-alone intervention	45.70 (11.30)	65.6	yes	yes

Zwerenz (2017) [97]	Germany	611	1) Blended treatment (inpatient face-to-face sessions plus internet-based CBT), 2) Active control group (inpatient face-to-face sessions plus online information on depression)	yes / yes	Secondary care (psychosomatic clinic)	Recurrent MDD (65.94%), single MDD (31.88), depression severity: mild (0.01%), moderate (79.91%), severe (16.59%) according to ICD-10 classification.	Acute treatment	Blended treatment	47.98 (9.79)	60.7	yes	no
Zwerenz (2017b) [31]	Germany	82	1) Psychodynamic web-based intervention plus mindfulness exercises<br>2) waiting list	yes / yes	Secondary care (after inpatient or day clinic psychotherapy)	Recurrent MDD (44.93%), single MDD (33.34), dysthymia (5.80); depression severity: mild (4.35%), moderate (56.52%), severe (17.39%) according to ICD-10 classification	Aftercare	Stand-alone intervention	ND	71	yes	no
**13 further published trials were identified in the course of the search update (August 2020) in the Cochrane Central Register of Controlled Trials. Publications are listed below**.												
Dennis (2020) [98]	Canada	241	1)Telephone interpersonal psychotherapy + locally available standard postpartum care (including postpartum depression service), 2) locally available standard	yes / no	Institutional (public health departments) and community (advertisement)	MDD (postpartum)	Acute treatment	Enhanced stand-alone intervention	ND	100	yes	no

			postpartum care (including postpartum depression service)								
Flygare (2020) [99]	Sweden	95	1) Internet-based CBT, 2) online psychoeducation control condition	no / no	Primary and secondary care	MDD (71.6%), dysthymia (9.5%), Depressive Disorder NOS (4.2%), MDD in partial remission (15.8%)	Acute treatment	Stand-alone intervention	45.30 (12.20)	75.80	yes	yes
Gili (2020) [100]	Spain	221	1) Online healthy lifestyle program, 2) online mindfulness program, 3) online positive affect promotion, 4) improved TAU	yes / yes	Primary care	MDD or dysthymia	Acute treatment	Enhanced stand-alone intervention	ND	77.8	no	yes
Hur (2018) [101]	South Korea	48	1) App-based cognitive restructuring, 2) App-based mood chart control group	no / no	Secondary care (outpatient psychiatric clinics) and community (advertisement)	Other specified depressive disorder	Acute treatment	Stand-alone intervention	ND	88.2	yes	no

Jannati (2020) [102]	Iran	78	1) App-based CBT, 2) waiting list	yes / no	Community (online annoucements)	MDD (postpartum)	Acute treatment	Stand-alone intervention	ND	100	yes	no
Johansson (2019) [108]	Sweden	54	1) Internet-based CBT, 2) waiting list	no / no	Secondary care (referral from psychiatric hospital)	Recurrent MDD (94%)	Acute treatment	Stand-alone intervention	ND	57.0	yes	no
Kooistra (2019) [103]	Netherlands	103	1) Blended CBT, 2) f2f CBT	yes / yes	Secondary care (routine specialized mental health care)	MDD	Acute treatment	Blended treatment	ND	63.0	yes	yes

Mohr (2019) [33]	USA	312	1) Stepped care program (1st step iCBT, 2nd step telephone CBT), 2) telephone CBT alone	yes / no	Community (advertisement) and health care system data (electronic medical records)	MDD	Acute treatment	Stepped care approach	ND	73.4	yes	yes
Oehler (2020) [104]	German y	348	1) Internet-based CBT, 2) progressive muscle relaxation control group	yes / yes	Community (advertisement)	Recurrent MDD (92.51%), single MDD (1.72%), dysthymia (23.92%)	Acute treatment	Stand-alone intervention	ND	78.7	yes	no
Pfeiffer (2020) [105]	USA	330	1) Peer-supported cCBT, 2) enhanced usual care	yes / no	Institutional (veterans affairs medical center and two of their associated outpatient clinics)	MDD	Acute treatment	Enhanced stand-alone intervention	51.6 (14.9)	20.0	yes	no

Pihlaja (2020) [106]	Finland	100	1) iCBT plus add-on telephone support, 2) iCBT only	yes / no	Recruitment from another trial	MDD	Acute treatment	Stand-alone intervention	63.11 (11.10)	66.00	no	yes
Richards (2020) [34]	UK	169	1) Immediate iCBT (step 2 within a stepped care approach), 2) waiting list	yes / yes	Routine care	MDD	Acute treatment	Stepped care approach	ND	ND	no	no
Welch (2019) [107]	USA	14	1) eCBT plus transcranial direct current stimulation, 2) eCBT plus sham stimulation	no / no	Community (advertisement) and clinician referrals	MDD	Acute treatment	Stand-alone intervention	53.21 (12.48)	85.71	no	yes

Note. N = Number of participants randomized; M = Mean; SD = Standard deviation; MDD = Major depressive disorder; ND = No data; PHQ-9 = Patient Health Questionnaire 9 item; BDI = Beck Depression Inventory; CBT = Cognitive behavioral therapy; TAU = treatment as usual; NOS = Not otherwise specified; GP = General practitioner; iCBT = Internet-based cognitive behavioral therapy; GAD = Generalized anxiety disorder; BA = behavioral activation; f2f = face-to-face.
