# Supplementary figures and images for: Evidence on Technology-Based Psychological Interventions in Diagnosed Depression: Systematic Review
Source: JMIR Ment Health. 2021 Feb 10;8(2):e21700. doi: 10.2196/21700 (PMC7904404; doi:10.2196/21700)

**Appendix 2.** Risk of bias assessment across all included studies (N=83).

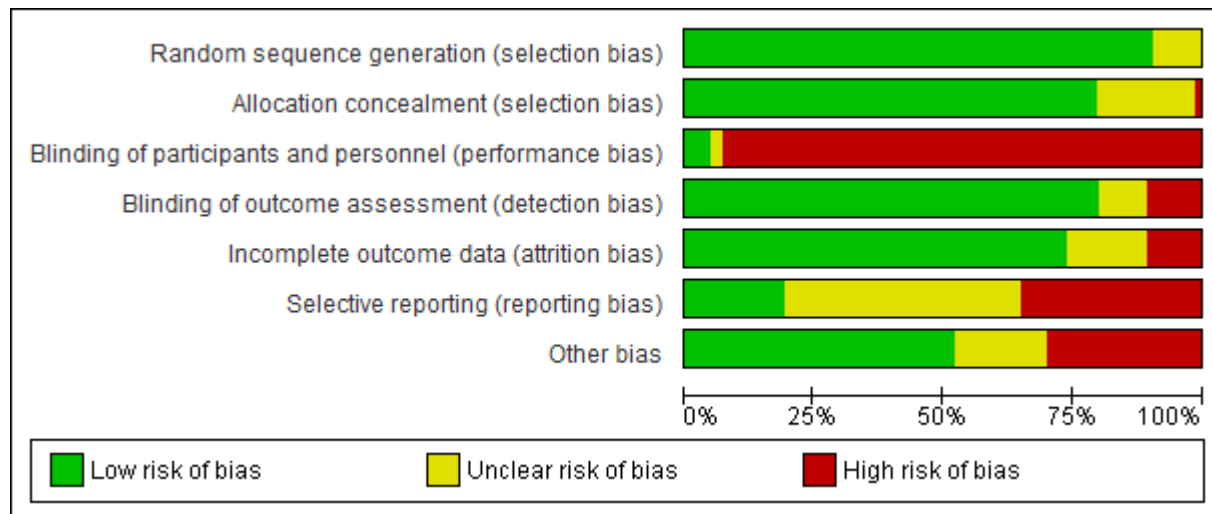

Supplement: Multimedia Appendix 2 [file mental_v8i2e21700_app2.pdf]
