# Supplementary material for: Evidence on Technology-Based Psychological Interventions in Diagnosed Depression: Systematic Review
Source: JMIR Ment Health. 2021 Feb 10;8(2):e21700. doi: 10.2196/21700 (PMC7904404; doi:10.2196/21700)
Supplement: Multimedia Appendix 3 [file mental_v8i2e21700_app3.pdf]

Appendix 3. Treatment characteristics of TBIs.

Study ID (Author / Year / Reference)	Name of TBI program	Study arms	Technical aspects of TBIs	Interventions' duration	Amount and role of human support in TBIS				Theoretical background
			**Applied technologies**	**Interventions' duration (in weeks) and planned number of modules / sessions**	**Degree of guidance[a]**	**Time of therapist's guidance** (any information provided by authors)	**Purpose of therapist's guidance**	**Qualification of therapists**	**Therapeutic rationale of TBIs**
Agyapong (2017) [35]	ND	1) Supportive text messages<br>2) control text messages	Internet application on mobile phone	13.05 weeks / two text messages per day for 90 days	Self-administered therapy	Self-administered therapy	Self-administered therapy	Self-administered therapy	CBT
Andersson (2013) [36]	ND	1) internet-based cognitive behavioral therapy<br>2) in-person group cognitive behavioral therapy	Internet-delivered text modules	8 weeks / 7 modules	Predominantly self-help	37.9 min per patient in total (SD = 11.5)	providing personalized feedback to homework	students at their last term of the clinical psychology program (five-year M.Sc.)	CBT
Arjadi (2018) [37]	Guided Act and Feel Indonesia (GAF-ID)	1) Internet-based behavioral activation intervention<br>2) online psychoeducation	Online program (accessible via computer or mobile device) + telephone support	ND / 8 modules	Predominantly self-help	150 min of telephonic support per person in the intervention arms	Explaining assignments, providing Feedback, reminding to log in	lay counsellors	BA

Berger (2011) [38]	Deprexis	1) Guided internet-based CBT<br>2) unguided internet-based CBT<br>3) waiting list	1) & 2), Online intervention	10 weeks / 11 modules	1) Predominantly self-help, 2) Self-administered therapy	1) ND, 2) Self-administered therapy	1) providing feedback, 2) Self-administered therapy	1) master of science students, 2) Self-administered therapy	1) & 2), CBT
Berger (2018) [39]	Deprexis	1) regular f2f psychotherapy plus internet-based CBT (Deprexis)<br>2) regular f2f psychotherapy	Online program	12 weeks (length of Deprexis) / ND	Blended treatment	Blended treatment	Blended treatment	Blended treatment	CBT
Blackwell (2015) [40]	ND	1) imagery cognitive bias modification<br>2) control condition (without training contingency, which was provided for other intervention arm)	Online program (access via web browser)	4 weeks / 12 sessions	Predominantly self-help	ND	brief overview of the intervention; standardized introduction to the intervention; guidance with practice session; help plan the sessions; monitor the adherence/ send emails to remind/ phone calls if someone missed a session	researchers guided participants	CBM
Bowers (1993) [41]	Overcomin g Depression	1) TAU<br>2) TAU plus therapist delivered CBT<br>3) TAU plus computer assisted CBT	3) Computer program	ND / 8 sessions	Blended treatment	Blended treatment	Blended treatment	Blended treatment	CBT
Carlbring (2013) [42]	Depression shjälpen	1) Computer-administered therapy<br>2) waiting list	1) Online intervention, CD-ROM (mindfulness and acceptance instructions exercises)	8 weeks / 7 modules	Minimal-contact therapy	The mean time per participant was 11.85 min a week, and the total time […] (M	providing feedback (validating, reinforcement of progress, encouraging to continue with intervention)	clinical psychology M.Sc. Students	1) BA with influenc es from accepta nce and commit

						= 94.8; SD = 33.5)			ment therapy
Celano (2017) [43]	ND	1) Positive psychology<br>2) cognition focused condition	Telephone	6 weeks / 6 sessions	Predominantly therapist-administered intervention	30 minutes telephone calls per person per week for 6 weeks	Review of exercise, assignment of next exercise, monitoring of suicidal thoughts	psychiatrists, psychologists, or licensed social workers trained by principal investigator on both conditions	PP, CF (cogniti on focused emotion ally neutral memory recall)
Choi (2012) [44]	Brighten Your Mood Program	1) Internet-based CBT (attuned to Chinese people with depression living in Australia)<br>2) waiting list	1) Online intervention, telephone (for supporting contacts)	8 weeks / 6 online	Minimal-contact therapy	The mean total support personnel time per Treatment group participant was 97.32 min (SD = 60.76 min) including sending and reading emails, and telephoning participants.	Reinforcement of progress; a summary of the key skills described in the Lesson for that week; normalizing difficulties with treatment; and encouragement to continue with the program.	clinicians	1) CBT

Choi (2014) [45]	ND	1) Videoconferencing problem solving therapy<br>2) face-to-face problem solving therapy, 3) telephone support calls	1) Laptop (Skype), 3) telephone	ND / 6 sessions	1), 3) Predominantly therapist-administered intervention	1) ND, 2) 6 calls (30 minutes) per person	1) providing problem solving therapy, 3) providing human contact for control group	1), 3) licensed master's-level social workers	1) Problem solving therapy, 3) no specific rationale applied
Corruble (2016) [46]	ND	1) Social rhythm therapy plus face-to-face treatment with a psychiatrist and antidepressant medication<br>2) clinical management plus face-to-face treatment with a psychiatrist and antidepressant medication<br>3) TAU (posteriori matched control group)	1) & 2), Telephone	1) 8 weeks / 8 sessions, 2) 8 weeks / ND	1) & 2), Blended treatment	1) & 2), Blended treatment	1) & 2), Blended treatment	1) & 2), Blended treatment	1) Social rhythm therapy, 2) Intensive clinical management (as described in the paper)
Egede (2015) [47]	ND	1) Videoconferencing behavioral activation treatment<br>2) face-to-face behavioral activation treatment	1) Videophone	8 weeks / 8 sessions	Predominantly therapist-administered intervention	Patients had 60 min sessions about once a week.	Providing psychotherapy via videophone	masters-level counsellors	2) Behavioral activation

Forand (2018) [48]	Beating the Blues v2.5 (plus TeleCoach)	1) Internet-based CBT<br>2) waiting list	1) Online Intervention, telephone (contacts with coaches)	8 weeks / 8 sessions	Predominantly self-help	Coaches spent about 1 to 2 hours in total working with each participant.	building reciprocity in the coaching relationship,<br>informing the user about expected behaviors,<br>setting expectations for progress, building expectations for accountability, and rewarding adherence	coaches (not further specified)	1) CBT
Forsell (2017) [49]	ND	1) Internet-based CBT plus antenatal TAU<br>2) antenatal TAU	Online platform	10 weeks / 10 modules	Minimal-contact therapy	Therapist spent on average a total of 2 h and 30 min (SD = 1 h and 50 min, range 35 min to 6,5 h) per patient over the course of the 10 weeks.	providing regular feedback, encouragements and support in written messages mirroring the interventions	CBT-trained therapist	CBT

Gilbody (2016) [50]	Beating the Blues; MoodGYM	1) Internet-based CBT (Beating the Blues) plus usual GP care<br>2) internet-based CBT (MoodGYM) plus usual GP care),<br>3) usual GP care	1) & 2), Online intervention, telephone (for technical support)	1) ND / 8 sessions, 2) ND / 5 sessions	1) & 2), Predominantly self-help	1) & 2), The total mean number of minutes of technical support calls delivered to participants was 6.2 (2-8) minutes for Beating the Blues participants and 6.5 minutes (2-9) for MoodGYM participants.	1) & 2), Encouragement to engage with intervention, answering questions related to technical issues	1) & 2), Trained technicians	1) & 2),
Graaf (2009) [51]	Colour Your Life	1) Internet-based CBT plus TAU<br>2) internet-based CBT<br>3) TAU	1) & 2), Online intervention	1) & 2), ND / 9 sessions	1) & 2), Self-administered therapy	1) & 2), Self-administered therapy	1) & 2), Self-administered therapy	1) & 2), Self-administered therapy	1) & 2), CBT
Holländare (2011) [16]	ND	1) Internet-based CBT<br>2) possibility of non-specific e-mail support	Internet-based	10 weeks / 9 (basic)and 7 (advanced) modules	Minimal-contact therapy	The mean number of messages from a therapist to a CBT participant was 15.3 (SD 6.3) (range 3–33) at an estimate of 10 min ⁄ message, resulting in	personalized feedback regarding homework and prompts when inactive	clinical psychologists and psychology students after completion of clinical training	CBT

						approximately 2.5 h of total therapist time / participant.			
Hunkeler (2012) [52]	eCare for Moods	1) Collaborative care website plus usual specialty mental health care 2) usual specialty mental health care	1) Online platform	52 weeks / ND	Predominantly self-help	ND	reviewing activity, using secure messaging, and responding to alerts automatically generated by participants' answers to monitoring questions	psychiatric nurse care manager (eCare manager)	CBT
Johansson (2012) [53]	SUBGAP	1) Internet-based psychodynamic treatment 2) structured support control group	1) Online intervention	10 weeks / 9 modules	Predominantly self-help	As expected, the average therapist time per participant and week was larger in the treatment group compared to the support group (13.2 minutes compared to 4.5 minutes, t(90) = 8.57, p<001). […]	providing feedback, administering gradual access to modules	final-semester students from a five-year M.Sc. clinical psychologist programme	Psychodynamic therapy
Johansson (2012) [54]	ND	1) Tailored internet-based CBT 2) standardized internet-based CBT 3) participation in online discussion	1) & 2), Online Intervention	1) 10 weeks / 25 chapters, 2) 10 weeks / 8 chapters	1) Minimal-contact therapy, 2) predominantly self-help	1) & 2), As expected, since most participants from the tailored	1) & 2), providing feedback on exercise, answering questions	1) & 2), MSc-level clinical psychologist	1) & 2), CBT

		group during waiting period				treatment received more modules, the average therapist time per participant was larger in the tailored group (95.2 minutes) compared to the standardized group (74.1 minutes).			
Johansson (2013) [55]	ND	1) Internet-based psychodynamic treatment<br>2) waiting list plus continuous contact with therapist	1) Online intervention<br>2) online contact to therapist	1) 10 weeks / 8 modules, 2) ND / ND	1) Minimal-contact therapy, 2) predominantly self-help	1) In the treatment group, the average therapist time per client and week was 9.5 min (SD D = 4.0), 2) The average therapist time per client and week was 2.3 min (SD = 0.86) in the control group.	1) providing feedback, administering gradual access to modules, 2) providing basic support (empathic listening, asking further questions) without applying specific techniques, symptom monitoring, questions on clients' experiences from previous week	1) & 2), Master's level students in their last semester of a 5-year clinical psychologist program	1) Psychodynamic therapy, 2) basic support from therapists

Kenter (2016) [32]	Alles onder Controle	1) Internet-based problem solving therapy<br>2) waitlist plus self-help book	Online intervention platform	5 weeks / 5 sessions	Predominantly self-help	15 to 20 min per person per session	nontherapeutic feedback helping participants to become familiar with techniques	masters-level students	PST (using self-examination therapy as a general framework)
Kessler (2009) [56]	ND	1) Online CBT plus usual GP care<br>2) usual GP care while on waitlist	Online intervention (with a therapist online in real time)	16 weeks / 10 sessions	Predominantly therapist-administered intervention	The intervention comprised up to ten sessions, each of 55 min of CBT delivered online, to be completed within 16 weeks of randomization when possible.	delivery of psychotherapeutic CBT sessions	CBT-trained psychologists	CBT

Kivi (2014) [57]	Depression shjälpen	1) Internet-based CBT<br>2) TAU	Online program, CD (containing mindfulness and acceptance instructions), telephone (contact to coach)	12 weeks / 7 modules	Minimal-contact therapy	The therapists were instructed to spend about 15 minutes by secure e-mail and/or telephone with each patient per week // 12x15 =180 minutes	take the role of the 'coach', focus feedback on validating the patient, reinforcing progress, and encouraging the patient to continue working with the program	licensed psychologists or psychotherapists	CBT (consisting of BA, Acceptance and Commitment Therapy, and Mindfulness)
Kok (2015) [29]	ND	1) Cognitive therapy plus Mood Monitoring plus TAU<br>2) TAU	Online intervention, telephone (contact to therapist)	ND / 8 sessions	Predominantly self-help	The mean amount of total time per therapist per participant during the complete mobile CT was 18 min (SD = 8.2).	Therapist support was aimed at working through the modules and finishing the therapy.	ND	CT
Hirsch (2018) [58]	ND	1) Online Cognitive Bias Modification with or, 2) without priming of repetitive negative thinking<br>3) neutral task control condition	Online platform	3 weeks / 10 sessions	Predominantly self-help	one in-person visit with participants (pre-training) and subsequent contact	Researchers provided instructions for completing the task at home.	ND	CBM
Lam (2013) [59]	ND	1) Telephone CBT plus medication	1) Telephone	8 to 10 weeks CBT; 12 weeks	Blended treatment	Blended treatment	Blended treatment	Blended treatment	CBT

		2) medication plus adherence reminder		escitalopram / 8 sessions of CBT					
Lang (2012) [60]	ND	1) Imagery-focused cognitive Bias modification<br>2) control condition with same stimulus material, but with modified contingencies	Computer program	1 weeks / 7 sessions	Self-administered therapy	Self-administered therapy	Self-administered therapy	Self-administered therapy	Imagery-focused CBM
Lappalainen (2015) [61]	Good Life Compass	1) Internet-based acceptance and commitment therapy<br>2) waiting list	Online intervention	7 weeks / 6 modules	Predominantly self-help	Coaches were instructed to keep the weekly feedback short, preferably within about 15 min per week	providing written feedback in order to encourage participants to exercises and to give suggestions	Master's-level student coaches	Acceptance and commitment therapy
Lindner (2014) [62]	Depression shjälpen	1) Self-help program plus telephone support by therapist<br>2) Self-help program plus e-mail support by therapist	1) Online program, telephone (contact to therapist), CD-ROM (with mindfulness and acceptance instructions)<br>2) Online program, e-mail (contact to therapist), CD-ROM (with	8 weeks / 7 modules	Predominantly self-help	15 min/ week (approx.) per participant	Validating the participant's experiences, providing feedback on performed tasks and reinforcing progress and plans of future work	MSc students in clinical psychology	BA (with some influence from acceptance and commitment therapy)

			mindfulness and acceptance instructions)						
Löbner (2018) [63]	MoodGym	1) Online CBT plus TAU<br>2) TAU	Online program	ND / 5 modules	Self-administered therapy	Self-administered therapy	Self-administered therapy	Self-administered therapy	CBT
Luxton (2016) [64]	ND	1) In Home treatment<br>2) in-person treatment	1) Videoconferencing via laptop	8 weeks / 8 sessions	Predominantly therapist-administered intervention	50-60-min sessions every week for 8 weeks per person	therapy via videoconferencing tools	doctoral-level mental health providers who received training from a BATD expert and consultant	BA
Ly (2014) [65]	ND	1) Smartphone-based BA intervention<br>2) smartphone-based mindfulness intervention	1) Smartphone application<br>2) smartphone application	8 weeks / 3 chapters	1) & 2), Minimal-contact therapy	1) & 2), The therapists reported a span between 2 and 18 min/week and participant.	1) & 2), encouraging messages, general educational messages + personal feedback on reflections + feedback on activities/ exercises	1) & 2), final-semester students from a 5-year MSc clinical psychologist programme	1) BA, 2) Mindfulness practice programme
Ly (2015) [66]	ND	1) Blended treatment (face-to-session plus smartphone application that were used between the sessions)<br>2) Face-to-face BA	1) Smartphone application	1) 9 weeks / 4 face-to-face sessions (smartphone app was used between	Blended treatment	Blended treatment	Blended treatment	Blended treatment	1) & 2), BA

				sessions),<br>2) 10 weeks / 10 sessions					
Mantani (2017) [67]	Kokoro-app	1) Smartphone CBT plus medication change<br>2) medication change alone	1) Smartphone application	9 weeks / 8 sessions	Blended treatment	Blended treatment	Blended treatment	Blended treatment	CBT
Meyer (2015) [68]	Deprexis	1) Internet-based CBT plus permission of using TAU<br>2) TAU	1) Online intervention plus daily text messages (optionally)	13.05 weeks / ND	Self-administered therapy	Self-administered therapy	Self-administered therapy	Self-administered therapy	CBT
Milgrom (2016) [69]	MumMood Booster	1) Internet-based CBT<br>2) TAU	1) Online intervention	8 weeks / 6 sessions	Minimal-contact therapy	(Coaches were instructed to spend a maximum of 30 minutes per week per participant). Of the six scheduled coach calls, participants completed a mean 4.3 calls (SD 2.2, range 0-6).	providing assistance and encouragement in the use and practice of particular strategies	coaches were graduate psychology trainees, clinical psychologists, and a health psychologist	CBT

Mohr (2011) [70]	ND	1) Telephone CBT<br>2) TAU	1) Telephone	20 weeks / 16 sessions	Predominantly therapist-administered intervention	T-CBT consisted of 16 sessions (45–50 min in length) provided over 20 weeks, allowing 4 weeks for missed sessions.	delivery of CBT via telephone	trained cognitive behavioral therapists	CBT
Mohr (2012) [71]	ND	1) Face-to-face CBT<br>2) Telephone CBT	2) Telephone administered intervention	18 weeks / 18 sessions	Predominantly therapist-administered intervention	Maximum time: 18(sessions) x 45 minutes = 810 mins	delivery of CBT via telephone	PhD level psychologists	CBT
Mohr (2013) [72]	MoodManager	1) Guided internet-based CBT<br>2) unguided internet-based CBT<br>3) waiting list	1) Online intervention, telephone (contact to coach)<br>2) online intervention	1) & 2), 12 weeks / 18 lessons	1) Predominantly self-help, 2) self-administered therapy	1) The mean engagement session time was 43.6 min, SD = 14.7, while the mean time for follow-up sessions was 8.9 (SD = 4.5) minutes, 2) self-administered therapy	TeleCoaching focused on enhancing adherence to moodManager by establishing a supportive relationship, setting and reviewing login goals, positively reinforcing login and site use, encouraging use of moodManager when login goals were not met, and answering any questions regarding the functionality of the site, 2) self-administered therapy	masters level social workers, PhD level psychologists, 2) self-administered therapy	1) & 2), CBT

Montero-Marin (2016) [73]	Smiling is Fun	1) Self-guided online program plus improved TAU<br>2) therapist-guided online program plus improved TAU<br>3) improved TAU	1) Online intervention<br>2) online intervention	1) & 2),: 13.05 weeks (estimated) / 10 module	1) Self-administered therapy, 2) Predominantly self-help	1) Self-administered therapy, 2) max. 3 e-mails in total (per person)	1) Self-administered therapy, 2) answering of questions and providing advice	1) Self-administered therapy, 2) trained psychotherapists	1) & 2), CBT
Nakao (2018) [74]	Kokoro (web-based CBT programme)	1) Blended CBT plus continuation with TAU with their treating psychiatrist<br>2) waiting list and continuation with TAU with their treating psychiatrist	1) Online program	12 weeks / 5 core components	Blended treatment	Blended treatment	Blended treatment	Blended treatment	CBT
Nyström (2017) [75]	ND	1) Physical activity without rationale<br>2) physical activity with rationale<br>3) BA based on Lewinshon's model<br>4) BA based on Martell's model<br>5) waiting list	3)-4) Online intervention	3)-4) 12 weeks / 8 sessions	3)-4) Predominantly self-help	3)-4) therapists were instructed to devote no more than 15 min per week per person	providing feedback	students from the Clinical Psychologists program	3)-4) BA
O'Mahen (2014) [76]	Netmums Helping With Depression (Netmums HWD)	1) BA<br>2) TAU	1) Online treatment, telephone (support)	ND / 12 sessions	Minimal-contact therapy	The average total time of sessions per participant was 253 min	answering questions about treatment material and working through barriers to treatment implementation	mental health workers with undergraduate degrees	BA

Perini (2009) [17]	Sadness Program	1) Online CBT<br>2) waiting list	1) Online intervention	8 weeks / 6 sessions	Minimal-contact therapy	111 min per participant	reinforcement for participation and efforts, encouragement to practice the treatment skills/to complete homework, enquiries about progress, response to questions	clinical psychologist	CBT
Reins (2019) [77]	GET.ON Mood Enhancer (shortened version)	1) Internet-based CBT<br>2) online psychoeducation	1)Online intervention	ND / 6 sessions	Minimal-contact therapy	on average 2 hours per participant	individualized written feedback after each module	psychotherapists-in-training	CBT
Ren (2016) [78]	MoodGym (Chinese version)	1) Internet-based CBT<br>2) waiting list	1) Online intervention	3 weeks / 5 sessions	Self-administered therapy	Self-administered therapy	reminder to complete modules	research assistants	CBT
Richards (2013) [79]	ND	1) Collaborative care intervention plus TAU<br>2) TAU	1) Telephone	14 weeks / 6 to 12 contacts	Predominantly therapist-administered intervention	The mean total time in collaborative care was 3.03 hours (standard deviation 2.18) over a period of 12 weeks (7.75).	depression education, drug management, behavioral activation, relapse prevention, and primary care liaison	mental health workers in primary care	BA
Rollman (2018) [80]	Beating the Blues	1) Internet-based CBT supported by collaborative care manager<br>2) Internet-based CBT supported by collaborative care manager plus access	1) Online intervention, telephone (for care managers contact)<br>2) Online intervention, telephone (for	1) & 2), 26.1 weeks / 8 sessions	1) & 2), Predominantly self-help	1) & 2), ND	1) & 2), review the program, establish rapport, monitor progress, send personalized feedback and encouragement via email/ call	college graduates with mental health research experience	1),2) CBT

		to internet support group<br>3) TAU	care managers contact),						
Rosso (2017) [81]	Sadness Program (adapted to American population, because it is originally from Australia)	1) Internet-based CBT<br>2) monitored attention control	1) Online intervention, telephone (for supporting contacts)	10 weeks / 6 lessons	Predominantly self-help	weekly 3-5 min calls per person	provide encouragement and support	bachelor-level research assistant	CBT
Sandoval (2017) [82]	ND	1) Interactive media-based, computer-delivered depression treatment program,<br>2) no treatment control condition	1) Computer-delivered intervention (at study site)	6 weeks / 6 sessions	Self-administered therapy	Self-administered therapy	Self-administered therapy	Self-administered therapy	PST
Schlicker (2018) [30]	ND	1) Standardized text message intervention<br>2) individualized text messages, 3) waiting control	1) Text messages via mobile phone,<br>2) text messages via mobile phone	1) & 2), 6 weeks / 81 text messages	1) & 2), Self-administered therapy	1) & 2), Self-administered therapy	1) & 2), Self-administered therapy	1) & 2), Self-administered therapy	1) & 2), ART (affect regulation training)
Schuver (2016) [83]	ND	1) Yoga intervention<br>2) walking control	DVD (for Yoga or walking, telephone (for contact to counsellors)	12 weeks / 8 sessions	Predominantly self-help	theoretical maximum of (8*15min) 120 min of therapist guidance	addressing questions and concerns about the DVD, setting goals, introducing and discussing current mindfulness topic	ND	Mindfulness-Bases Stress Reduction (MBSR)

Selmi (1990) [84]	ND	1) Computer-administered therapy<br>2) therapist-administered therapy<br>3) waiting list	1) Computer-delivered intervention (at study site)	6 weeks / 6 sessions	Predominantly self-help	The computer treatment group had some additional contact with the experimenter (about 20 minutes/week) for assessments.	In the computer condition the patients had only minimal contact with the experimenter, who helped start and end the sessions and, although not in the room while the patient was interacting with the computer, was available to answer questions. Most questions had to do with computer procedures, and contact was kept to a minimum	ND	CBT
Smith (2017) [85]	Sadness Program	1) Internet-based CBT<br>2) self-help book (CBT)<br>3) self-help book (meditation)<br>4) waiting list	1) Online intervention, optional email/telephone contact to technician/clinician	12 weeks / 6 modules	Predominantly self-help	The technician spent an average of 9.7 min (SD = 5.02, range =3–21) emailing and calling each participant in the iCBT group during the treatment course (including follow-up period).	Participants were contacted via email or telephone by research staff (‘The Technician’, JS) after the first two lessons, then as requested, or by a clinician (psychiatry registrar) in response to an increase in distress or suicidal intent.	ND	CBT

Steinmann (2020) [15]	ND	1) Telephone CBT with reminding letters<br>2) telephone CBT without reminding letters	1) Telephone-delivered<br>2) telephone delivered	9 to 13 weeks / at least 9 sessions	1) & 2), Predominantly therapist-administered intervention	1) & 2), approx. 30 minutes per session per person	1) & 2), provide psychotherapy by telephone	1) & 2), licensed CBT-psychotherapists	1) & 2), CBT
Thase (2018) [86]	Good Days Ahead (GDA)	1) Blended CBT treatment<br>2) face-to-face CBT	1) Online intervention	16 weeks / 9 modules (online) plus 12 face-to-face sessions	Blended treatment	Blended treatment	Blended treatment	Blended treatment	CBT
Titov (2010) [87]	Sadness Program	1) Internet-based CBT plus technician support<br>2) Internet-based CBT plus clinician support<br>3) waiting list	1) & 2), Online intervention, telephone (for clinician/technician contact)	1) & 2), 8 weeks / 6 lessons	1) & 2), Predominantly self-help	1) total time spent by the technician (mean, SD, 61.0 mins, 9.8) per person, 2) total time spent by clinician (mean, SD, 60.5 mins, 19.0) per person	1) Provide encouragement and support, respond to general questions by referring to the material of the program. No clinical advice, 2) active engagement in treatment, including goal setting, problem solving, discussion of strategies for overcoming hurdles to progress	1) no prior experience with research programs, no qualifications in health care or counseling, no clinical duties in her usual role, 2) qualified and registered psychiatrist	1) & 2), CBT

Titov (2011) [88]	Wellbeing Program	1) Internet-based CBT<br>2) waiting list	1) Online intervention, contact to clinician via instant messaging or telephone	10 weeks / 8 modules	Predominantly self-help	The mean total therapist time per Treatment group participant was 84.76 min (SD = 50.37 min) including monitoring of the discussion forum, sending and reading instant messages, and telephoning participants.	1) Reinforcement of progress; 2) a summary of the key skills described in the Lesson for that week and encouraging practice of homework tasks; 3) normalizing difficulties with treatment; 4) encouragement to continue with the program, and; 5) answering questions and providing feedback to participants about their use of the skills.	clinical psychologist	CBT
Torkan (2014) [89]	ND	1) Positive imagery cognitive bias modification<br>2) non-imagery control condition	1) & 2), Computer-delivered intervention	1 week / 7 sessions	Self-administered therapy	Self-administered therapy	Self-administered therapy	Self-administered therapy	1) CBM
Vernmark (2010) [90]	ND	1) Email therapy<br>2) internet-based CBT<br>3) waiting list	1) & 2), Online Intervention	1) & 2), 8 weeks / 8 modules	1) Predominantly therapist-administered intervention, 2) predominantly self-help	1) The total average time spent by each therapist was 509 min (SD =176, range:171–890) per participant,	1) providing psychotherapy by email, 2) providing passwords to text modules, reinforcement of progress	1) & 2), psychology MSc students	1) & 2), CBT

						2) Each therapist spent an average of 53 minutes per participant (SD = 28, range 10–165) for the whole self-help treatment.			
Watkins (2012) [91]	ND	1) Cognitive bias modification plus TAU, 2) relaxation training plus TAU<br>3) TAU	1)CD-ROM (with exercises), telephone	8 weeks / ND	Predominantly self-help	ND	during the telephone sessions the trainer provided feedback, guidance, and encouragement to ensure accurate use of the exercise, monitored progress and scheduled regular progress	graduate level psychologists or postdoctoral psychologists or clinical psychologist	CBM
Watts (2013) [92]	The Sadness Program (originally), but the title was changed to The Get Happy Program	1) Mobile-based intervention (via an application)<br>2) computer-based intervention	1) Access to intervention via app (mobile phone or iPad)<br>2) Access to intervention via computer	1) & 2), 8 weeks / 6 sessions	1) & 2), Predominantly self-help	1) & 2), The mean clinician time spent per participant was 4.1 minutes (SD= 4.63) and the mean technician time spent per participant was 6.4	1) & 2), ND	1) & 2), ND	1) & 2), CBT

						minutes (SD= 5.38)			
Williams (2013) [93]	Sadness Program in conjunction with cognitive bias modification (OxIGen = Oxford Imagery Generation)	1) imagery-focused cognitive bias modification plus internet-based CBT<br>2) waiting list	1) Online interventions (CBM, CBT)	11 weeks / 7 CBM sessions plus 6 CBT sessions	Self-administered therapy	Self-administered therapy	Self-administered therapy	Self-administered therapy	1) CBM, CBT
Williams (2015) [94]	CBM (the program is labelled OXIGen in the trial registration) plus Sadness Program	1) Positive imagery cognitive bias modification plus internet-based CBT<br>2) control cognitive bias modification condition plus internet-based CBT	1) & 2), Online interventions (CBM, CBT)	1) & 2), 11 weeks / 7 active/control CBM sessions plus 6 CBT sessions	1) & 2), Predominantly self-help	1) & 2), ND	1) & 2), addressing patients' queries	1) & 2), doctoral level psychologists	1) CBM plus CBT, 2) control CBM plus CBT
Wright (2005) [95]	ND	1) Blended cognitive therapy<br>2) face-to-face cognitive therapy<br>3) waiting list	1) Computer-delivered intervention (at study site)	8 weeks / 9 face-to-face session followed immediately by 8 computer sessions	Blended treatment	Blended treatment	Blended treatment	Blended treatment	1) Cognitive therapy (or CBT)

Zagorscak (2018) [96]	ND	1) Internet-based CBT plus standardized, automated feedback and contact on demand<br>2) internet-based CBT plus semistandardized e-mail feedback	1) & 2), Online intervention	6 weeks / 7 modules	1) Self-administered therapy, 2) Predominantly self-help	1) & 2), ND	1) answering on-demand messages, 2) written feedback after each completed module (personalized)	1) members of research team, 2) counselors holding either a bachelor's or master's degree in psychology	1) ,2) CBT
Zwerenz (2017) [97]	Deprexis	1) Blended treatment (inpatient face-to-face sessions plus internet-based CBT), 2) Active control group (inpatient face-to-face sessions plus online information on depression)	1) Online intervention	12 weeks / 12 modules	Blended treatment	Blended treatment	Blended treatment	Blended treatment	1) CBT
Zwerenz (2017) [31]	ND	1) Psychodynamic web-based intervention plus mindfulness exercises<br>2) waiting list	1) Online intervention	10 weeks / 10 modules	Predominantly self-help	ND	providing encouraging feedback	trained psychologist	1) Psychod ynamic therapy, mindful ness
**13 further published trials were identified in the course of the search update (August 2020) in the Cochrane Central Register of Controlled Trials. Publications are listed below**									
Dennis (2020) [98]	ND	1) Telephone interpersonal psychotherapy + locally available standard postpartum care (including postpartum depression service), 2) locally available standard postpartum	1) Telephone-delivered	12 weeks / 12 modules	1) Predominantly therapist-administered intervention	ND	providing interpersonal therapy via telephone	seven registered female nurses based in Toronto were trained to provide the IPT intervention.	1) IPT

		care (including postpartum depression service)							
Flygare (2020) [99]	ND	1) Internet-based CBT, 2) online psychoeducation control condition	1) Online intervention	8 weeks / 8 modules	1) Predominantly self-help	ND	The psychologist gave feedback on completed homework, answered questions and gave access to the next module in their replies. Feedback on homework in the ICBT-group were typically written to help the participant to understand and to use the strategies in behavioral activation and cognitive restructuring.	psychologists	1) CBT
Gili (2020) [100]	ND	1) Internet-based mindfulness program,2) internet-based positive affect promotion program 3) internet-based healthy lifestyle program, 4) improved TAU	1) & 2), Online intervention	4 to 8 weeks / 4 modules	1) & 2), Predominantly self-help	1) & 2), 90 f2f session at the beginning of the intervention	1) & 2), The aim of this session was to explain the program structure and main components of treatment and to motivate participants for change	ND	1) Mindfulness, 2) Positive affect promot-ion
Hur (2018) [101]	Todac Todac (TT)	1) App-based cognitive restructuring, 2) app-based mood chart control group	1) Online intervention	3 weeks / ND	1) Self-administered therapy	1) Self-administered therapy	1) Self-administered therapy	1) Self-administered therapy	1) CT (especially cognitive

									restructuring)
Jannati (2020) [102]	Happy Mom	1) App-based CBT, 2) waiting list	1) Online intervention	8 weeks / 8 modules	1) Self-administered therapy	1) Self-administered therapy	1) Self-administered therapy	1) Self-administered therapy	1) CBT
Johansson (2019) [108]	ND	1) Internet-based CBT, 2) waiting list	1) Online intervention	8 weeks / 8 modules	1) Predominantly self-help	ND	The therapist gave text-based feedback (within a 24-hour time frame) focusing on encouraging the participants work and keeping the participant focused on the program. If participants did not report their assignments, the therapist phoned the participants and attempted to problem-solve how participants could adhere better to the treatment protocol. If the assignment was not performed as expected the clinician made an assessment if to continue with the next weekly module or to repeat the previous.	two licensed psychologists	1) CBT

Kooistra (2019) [103]	ND	1) Blended CBT, 2) f2f CBT	1) Online intervention	10 weeks / 10 f2f sessions and 9 web-based sessions	Blended treatment	Blended treatment	Blended treatment	Blended treatment	1) CBT
Mohr (2019) [33]	ThinkFeel-Do	1) Stepped care program (1st step iCBT, 2nd step telephone CBT), 2) telephone CBT alone	1) Online intervention, 2) telephone-delivered intervention	1) & 2), duration of the treatment depended on treatment success, but treatment lasted up to a maximum of 20 weeks	1) Predominantly self-help, after stepping up the intervention was therapist-administered, 2) predominantly therapist-administered intervention	1) Coaches began with a 30–40 min engagement phone call, followed by weekly 10–15 min calls and 2–3 secure messages per week through the ThinkFeelDo site. After 3 weeks, patients could elect to move to messaging only or continuing to use telephone coaching on an as-needed basis.; 2) spoke weekly with their	1) The iCBT coaching protocol was manualized (Tomasino, Lattie, Wilson, & Mohr, 2017) and based on the supportive accountability and efficiency models (Mohr, Cuijpers, & Lehman, 2011; Schueller, Tomasino, & Mohr, 2016). […] The initial session will be conducted via telephone and will last about 30 - 45 minutes long in order to facilitate engagement with the coach and the program, with subsequent sessions of approximately 5-15 minutes per week. [...] Coaching sessions will follow a motivational interviewing based format focused on increasing participants' motivation to	1) & 2), 5 PhD level clinical psychologists, 2 licensed clinical social workers, and a master's level therapist, all of whom were trained CBT therapists.	1) & 2), CBT

						assigned therapist for 45–50 min.	continue logging in to the website.; 2) delivering CBT via telephone		
Oehler (2020) [104]	iFightDepression	1) Internet-based CBT, 2) progressive muscle relaxation control group	1) Online intervention	6 weeks / 6 modules	1) Predominantly self-help	The mean time spent on the 5 guidance and study calls was 38.5 (SD 9.9) min per participant for the iFD group and 28.9 (SD 15.0) min per participant for the PMR group, […]	The focus of the guidance calls was to motivate the participants rather than discuss the intervention content.	psychologists and psychotherapists	1) CBT
Pfeiffer (2020) [105]	Beating the blues	1) Peer-supported cCBT, 2) enhanced usual care	1) Online intervention	13 weeks / 8 modules	1) Predominantly self-help	ND	[…] to discuss progress and barriers to completing cCBT modules and to provide general peer support for managing depression.	trained and supervised peer support specialists	1) CBT
Pihlaja (2020) [106]	ND	1) iCBT plus add-on telephone support, 2) iCBT only	1) & 2), Online intervention	ND / 7 modules	1) & 2), Predominantly self-help	1) 73.37 (48.95), 5-165 => mean duration of phone calls, 2) 1.95 (6.11), 0-30 => mean	1) & 2), […]; patients receive encouragement to write to the therapist any time with their questions or concerns. The therapist comments on the completed tasks, offering praise and	1) & 2), All 5 therapists involved in this trial were clinical psychologists employed by HUS and had	1) & 2), CBT

						duration of phone calls	support for the patient. ;1)[...] In addition to the standard HUS-iCBT, the add-on STS intervention group […]selection of individual goals took place. The tasks and themes of each module were discussed in the calls that followed each module. Support was individually tailored and followed the principles of the model of supportive accountability [33], […] According to this model, combining elements of support and accountability with a legitimized and trustworthy relationship increases adherence.[…]	at least 2 years of work experience with depressed patients.	
Richards (2020) [34]	Space from Depression	1) Immediate iCBT (step 2 within a stepped care approach), 2) waiting list	1) Online intervention	8 weeks / ND	1) Predominantly self-help	PWPs were instructed to provide six online reviews (15 min per participant per review) over the 8-week intervention period.	During the reviews, PWPs provided feedback to clients based on their work from week-to-week (e.g. modules completed, tools used, shared journal entries) and encouragement in order to promote meaningful engagement	[…] PWPs, who are psychology graduates specifically trained in the provision of low intensity CBT interventions.	1) CBT

							with the programme.		
Welch (2019) [107]	Good Days Ahead (GDA)	1) eCBT plus transcranial direct current stimulation, 2) eCBT plus sham stimulation	1) & 2), Computer-delivered intervention (at study site)	1) & 2), 4 weeks / ND	1) & 2), Self-administered therapy	1) & 2), Self-administered therapy	1) & 2), Self-administered therapy	1) & 2), Self-administered therapy	1) & 2), CBT

Note. [a]Newman et al. [109]; ND = No data; SD = Standard deviation; CBT = Cognitive behavioral therapy; BA = Behavioral activation; CBM = Cognitive bias modification; PP = Positive psychology; CF = Cognition focused; CT = Cognitive therapy; PA = physical activity; PST = Problem solving therapy; IPT = interpersonal therapy; f2f = Face-to-face; TAU = Treatment as usual; M. Sc. = Master of Science.
