# Supplementary material for: Evidence on Technology-Based Psychological Interventions in Diagnosed Depression: Systematic Review
Source: JMIR Ment Health. 2021 Feb 10;8(2):e21700. doi: 10.2196/21700 (PMC7904404; doi:10.2196/21700)
Supplement: Multimedia Appendix 4 [file mental_v8i2e21700_app4.pdf]

Appendix 4. Technologies for intervention delivery in TBI arms (n=107).

**Descriptor of applied technical medium(s)**	**Number of identified TBI arms**	**Proportion (in %) on all TBI-arms**
**At least one interventional component was delivered via the internet**	**80**	**75**
Internet-based intervention	58	54
Internet-based intervention + telephone support	18	17
Internet-based intervention + CD-ROM + telephone support	2	2
Internet-based intervention + CD-ROM	2	2
**At least one interventional component was delivered via telephone (eg, telephone support)**	**34**	**32**
Telephone	12	11
Internet-based intervention + telephone support	18	17
Internet-based intervention + CD-ROM + telephone support	2	2
Telephone + DVD	1	1
CD-ROM + telephone	1	1
**Others**	**13**	**12**
Mobile phone (text messages)	2	2
Videoconferencing	3	3
Offline computer program	8	7

Note. Color highlighting of lines that were considered twice in different categories of applied technical mediums.
