# Supplementary material for: Evidence on Technology-Based Psychological Interventions in Diagnosed Depression: Systematic Review
Source: JMIR Ment Health. 2021 Feb 10;8(2):e21700. doi: 10.2196/21700 (PMC7904404; doi:10.2196/21700)
Supplement: Multimedia Appendix 5 [file mental_v8i2e21700_app5.pdf]

Appendix 5. Therapeutic rationales of TBI arms (n=107) in included studies.

**Therapeutic rationale of TBIs**	**Number of identified TBI-arms**	**Proportion (in %) on all TBI-arms***
Cognitive behavioral treatment approaches (behavioral activation [n=12], cognitive behavioral therapy [n=70], cognitive therapy [n=3])	85	79
Cognitive bias modification	7	7
Mindfulness	4	4
Problem solving therapy	3	3
Psychodynamic therapy	3	3
Affect regulation training	2	2
Acceptance and commitment therapy	1	1
Cognition focused treatment (ie, emotionally neutral memory recall)	1	1
Intensive clinical management	1	1
Interpersonal psychotherapy	1	1
Positive affect promotion	1	1
Positive psychology	1	1
Social rhythm therapy	1	1

Note. *Overall percentage exceeds 100%, since three trials combined rationales (eg, psychodynamic therapy and mindfulness), and were thus considered repeatedly.
