# Supplementary material for: Evidence on Technology-Based Psychological Interventions in Diagnosed Depression: Systematic Review
Source: JMIR Ment Health. 2021 Feb 10;8(2):e21700. doi: 10.2196/21700 (PMC7904404; doi:10.2196/21700)
Supplement: Multimedia Appendix 6 [file mental_v8i2e21700_app6.pdf]

Appendix 6. Report of negative events.

Study ID (author / year / reference)	Deterioration	Adverse events	Severe adverse events	Novel Symptoms	Dropout	Nonresponse	Unwanted events
Agyapong (2017) [35]							
Andersson (2013) [36]							
Arjadi (2018) [37]							
Berger (2011) [38]							
Berger (2018) [39]							
Blackwell (2015) [40]							
Bowers (1993) [41]							
Carlbring (2013) [42]							
Celano (2017) [43]							
Choi (2012) [44]							
Choi (2014) [45]							
Corruble (2016) [46]							
Egede (2015) [47]							
Forand (2018) [48]							
Forsell (2017) [49]							
Gilbody (2016) [50]							
Graaf (2009) [51]							
Holländare (2011) [16]							
Hunkeler (2012) [52]							
Johansson (2012) [53]							
Johansson (2012) [54]							
Johansson (2013) [55]							
Kenter (2016) [32]							
Kessler (2009) [56]							
Kivi (2014) [57]							
Kok (2015) [29]							
Hirsch (2018) [58]							

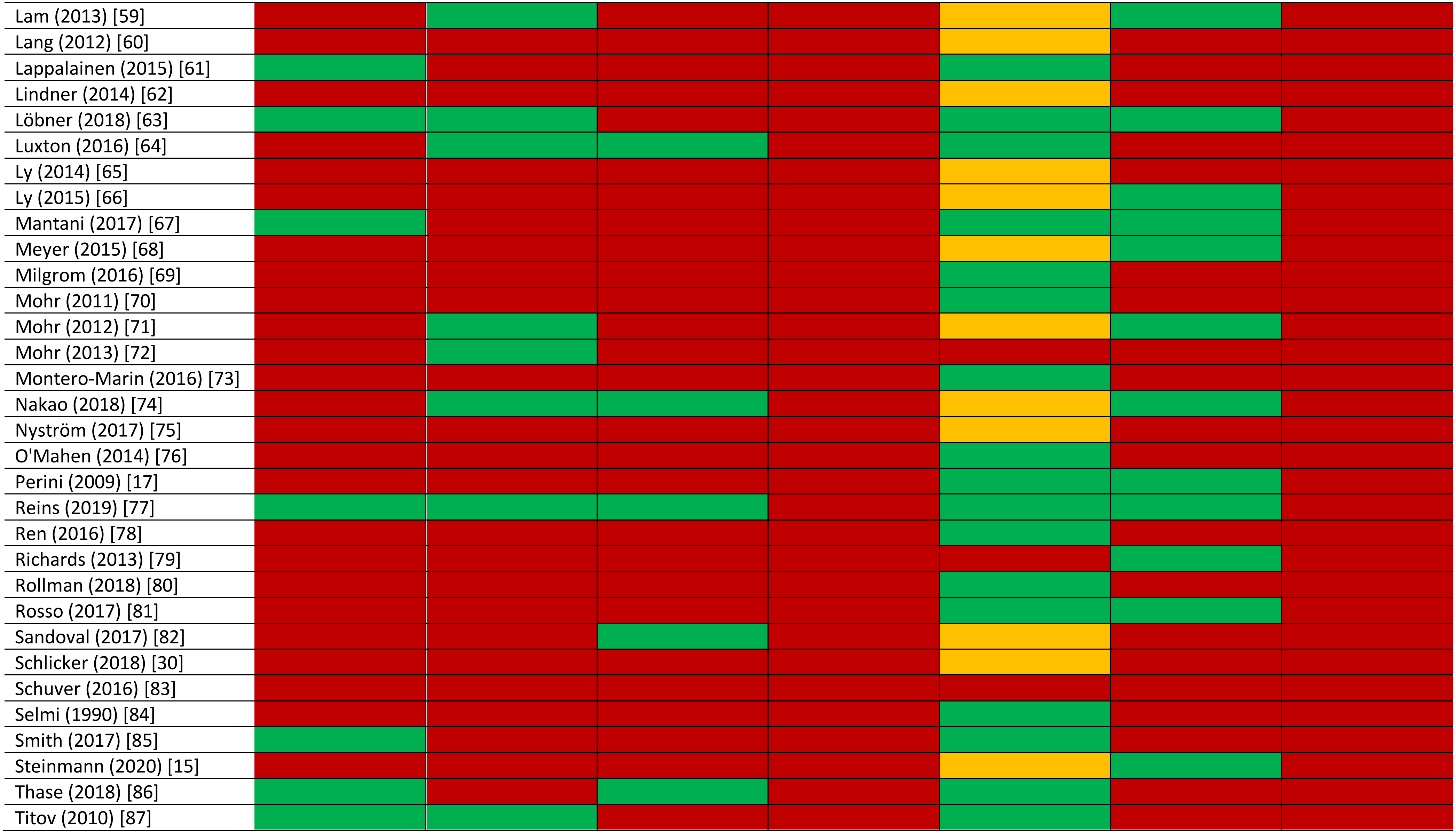
Lam (2013) [59]
Lang (2012) [60]
Lappalainen (2015) [61]
Lindner (2014) [62]
Löbner (2018) [63]
Luxton (2016) [64]
Ly (2014) [65]
Ly (2015) [66]
Mantani (2017) [67]
Meyer (2015) [68]
Milgrom (2016) [69]
Mohr (2011) [70]
Mohr (2012) [71]
Mohr (2013) [72]
Montero-Marin (2016) [73]
Nakao (2018) [74]
Nyström (2017) [75]
O'Mahen (2014) [76]
Perini (2009) [17]
Reins (2019) [77]
Ren (2016) [78]
Richards (2013) [79]
Rollman (2018) [80]
Rosso (2017) [81]
Sandoval (2017) [82]
Schlicker (2018) [30]
Schuver (2016) [83]
Selmi (1990) [84]
Smith (2017) [85]
Steinmann (2020) [15]
Thase (2018) [86]
Titov (2010) [87]

Titov (2011) [88]	green	red	red	red	yellow	red	red
Torkan (2014) [89]	red	red	red	red	yellow	red	red
Vernmark (2010) [90]	red	red	red	red	green	red	red
Watkins (2012) [91]	red	green	red	red	yellow	red	red
Watts (2013) [92]	red	red	red	red	green	red	red
Williams (2013) [93]	red	red	red	red	yellow	red	red
Williams (2015) [94]	red	red	red	red	green	red	red
Wright (2005) [95]	red	red	red	red	yellow	red	red
Zagorscak (2018) [96]	green	red	red	red	green	red	red
Zwerenz (2017) [97]	red	red	red	red	yellow	red	red
Zwerenz (2017) [31]	red	red	red	red	red	red	red
**13 further published trials were identified in the course of the search update (August 2020) in the Cochrane Central Register of Controlled Trials. Publications are listed below**							
Dennis (2020) [98]	red	red	red	red	yellow	red	red
Flygare (2020) [99]	red	red	red	red	green	red	red
Gili (2020) [100]	red	red	red	red	green	red	red
Hur (2018) [101]	red	red	red	red	red	red	red
Jannati (2020) [102]	red	red	red	red	red	red	red
Johansson (2019) [108]	green	red	red	red	green	red	red
Kooistra (2019) [103]	green	red	green	red	yellow	red	red
Mohr (2019) [33]	red	red	green	red	yellow	red	red
Oehler (2020) [104]	red	green	green	red	yellow	red	red
Pfeiffer (2020) [105]	red	red	red	red	red	red	red
Pihlaja (2020) [106]	red	red	green	red	green	red	red
Richards (2020) [34]	green	red	green	red	red	red	red
Welch (2019) [107]	red	green	red	red	yellow	red	red

Note. Color highlighting of cells indicates whether or not information concerning different categories of negative events were reported in included studies: green = report concerning information on specific category of negative events (see first line); red = no report concerning information on specific category of negative events (see first line); yellow (only applied for dropouts) = report of other kind of dropouts (eg, withdrawals from study, treatment completers as defined by authors) that did not capture dropouts from treatment (ie, inverse of the completion rate, etc.).
